# Civility as Politeness During COVID-19

**DOI:** 10.1007/978-981-33-6706-7_3

**Published:** 2021-03-03

**Authors:** Matteo Bonotti, Steven T. Zech

**Affiliations:** Politics and International Relations, Monash University, Melbourne, Australia

## Abstract

This chapter examines new challenges to the politeness dimension of civility presented by COVID-19. First, the pandemic has made it more difficult for people to identify norms of politeness and behave appropriately in circumstances that were previously less contested and problematic. Furthermore, signalling respect and consideration towards others via polite speech or behaviour is more likely to go awry during COVID-19. Additionally, the lack of clarity surrounding norms of politeness may prevent polite acts from helping to mitigate conflict and facilitate cooperative social exchange. Finally, citizens and politicians can exploit disruptions around politeness norms to engage in behaviour that under normal circumstances would be considered impolite. The chapter also identifies potential solutions that governments, businesses, and citizens can adopt to respond to these challenges.

## Introduction

This chapter examines new challenges to the politeness dimension of civility presented by COVID-19. As we explained in Chapter 10.1007/978-981-33-6706-7_2, politeness involves complying with norms of behaviour that aim to facilitate peaceful exchange and positive social interaction. At the very onset of the pandemic, compliance with politeness norms and other-regarding behaviour served as an early bulwark against the spread of the virus. As the director of the Division of Medical Ethics at New York University’s Grossman School of Medicine Arthur Caplan said, ‘[a]t least for now, we don’t have treatment or vaccines. All we’ve got is behaviour. And there is evidence that the behaviour works, if we’re diligent about it’.^1^ How people behave during a crisis such as a pandemic has very real consequences. In the absence of clear guidelines and policies, polite other-regarding behaviour can save lives.

However, the crisis resulting from the pandemic also disrupted politeness norms themselves. We focus on some of these disruptions in this chapter. More specifically, we identify four problems posed by the pandemic to people’s ability to behave politely. First, the current crisis has made it difficult for people to identify norms of politeness and behave appropriately in circumstances that were previously less contested and problematic. Second, the signalling function of politeness has also been undermined: communicating respect and consideration towards others via polite speech or behaviour is more likely to go awry during the pandemic. Third, new problems regarding social cooperation may arise as a result. When acts of politeness are unclear or misunderstood, they may no longer help to mitigate conflict or facilitate cooperative social exchange. Finally, citizens and politicians can exploit disruptions around politeness norms to engage in behaviour that under normal circumstances would be considered impolite.

## Navigating New Politeness Norms

As we explained in the previous chapter, behaving politely implies complying with social norms grounded in people’s social identities and roles, which are often affected by contextual and environmental factors. However, politeness also involves an agential dimension, namely the ability to be aware of, critically assess, judge, and comply with those norms based on the circumstances. In normal times, agents exercise this kind of critical engagement against the background of relatively stable politeness norms. However, COVID-19 has disrupted this stability and forced agents to navigate a social environment characterized by greater uncertainty, where it is more difficult to identify, evaluate, and comply with politeness norms. In this sense, critical engagement with norms of politeness has not only become increasingly difficult, but also more necessary during the current pandemic: when the structural dimensions of politeness are unstable or contested, agents are less likely to find and adopt ready-made polite behaviours. Instead, they need to make a greater effort to understand what behaving politely entails in different circumstances. Yet, people are endowed with the capacity to adapt to these disruptions and formulate solutions to problems raised by this kind of crisis. In this subsection we evaluate whether and how individuals or groups have responded to the disruption of politeness norms during the current pandemic.

In what ways has COVID-19 rendered critical judgment related to norms of politeness more problematic? It seems evident that people have often faced a lack of clear information regarding the virus, the way it spreads, and its short- and long-term effects—especially at the beginning of the pandemic. However, the problem persists due to limited or uncertain scientific evidence regarding how the virus spreads in everyday environments such as gyms, restaurants, cafes, or on public transport.^2^ This points to an ongoing challenge since many of our polite interactions with others happen in these kinds of spaces. For example, if it is unclear how the virus spreads within a restaurant, then it will be difficult for people to understand how to interact politely with their table companions, the waiting staff, or other customers; what in normal circumstances used to be considered polite (e.g. handing over a menu, passing the salt, or exchanging pleasantries with a neighbouring table) might no longer be viewed as such. Furthermore, and relatedly, in the absence of clear information people may end up making different judgments about what constitutes politeness and how to express it in different contexts and circumstances. This may risk creating different and separate ‘politeness bubbles’, thus undermining the ‘social lubricant’ function of politeness, a point to which we will return in more detail later in the chapter.

**Image 3.1:**
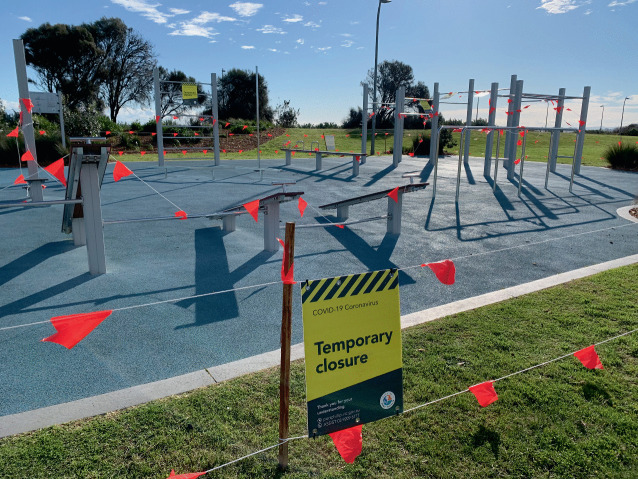
Roped off outdoor gym equipment in Melbourne, Australia

This ‘piecemeal’ approach to politeness, where people adopt different norms of politeness (and to different degrees) in the absence of clear information, constitutes one of the most significant challenges COVID-19 poses to the politeness dimension of civility. Responding to this situation requires interaction and communication between various agents within society. For example, governments might help to coordinate and disseminate findings from scientific research on the virus in order to provide citizens with clearer guidelines, including guidance regarding other-regarding behaviours that might constitute polite action in view of the current pandemic. This might involve issuing press releases as well as providing clear information about how to behave politely under the current circumstances in popular public locations such as beaches and parks. This might take the form of signs or ropes and fences to clearly deter use (Image 3.1). People already provide these kinds of information tools concerning etiquette for visiting mosques,^3^ Buddhist temples,^4^ or churches.^5^

In the remainder of this section, we will consider three examples regarding uncertain politeness norms: queuing, interactions in sites related to the food industry such as restaurants and cafes, and greetings. We will illustrate how COVID-19 has caused a disruption of politeness norms in all three contexts, and how citizens, businesses, and governments have reacted to this crisis in order to restore such norms.

### Queuing During COVID-19

Queuing provides a clear example of a politeness norm that is simple and universally understood as a polite act regardless of levels of compliance. While we may observe subtle variation in queuing practices across contexts, an Italian (for example) would understand how to stand in a line in the United States and vice versa. Yet, at the start of the pandemic, considerations related to the transmission of COVID-19 disrupted previous practices and added new layers of complexity to queuing. New demands emerged about maintaining physical distance in public places, but solutions came quickly. People acknowledged the need to make small changes to their behaviour and almost immediately began to help others understand new expectations. For example, in Seattle, Washington vendors at a local farmer’s market provided additional guidance with tape, chalk, and signs to help patrons adhere to new best practices.^6^

**Image 3.2:**
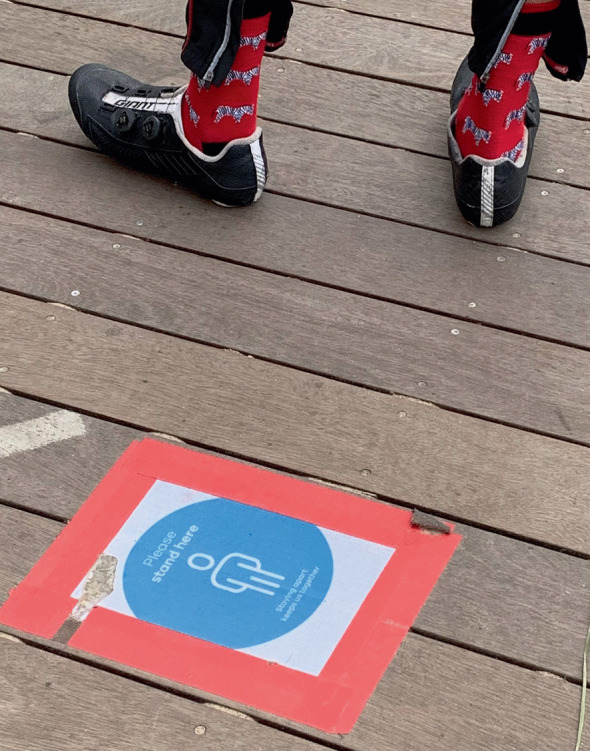
A business providing guidance on queuing practices in Melbourne, Australia

Explicit measures such as floor markings can communicate emerging politeness norms related to queuing—reducing uncertainty and making compliance with new norms more likely. The solutions may be formal or informal and the norms more or less codified. Some governments stepped in to provide guidance and a more authoritative stance on physical distancing. For example, the state government in Tasmania, Australia encouraged businesses to act: ‘[t]o maintain physical distancing, place appropriately spaced floor markings at queuing points, such as checkouts and the entrance to the store. At larger stores, queuing stations need to be clearly identified outside the store.’^7^ Supermarkets added markings and moved especially long queues to outside spaces.^8^ Some businesses adopted more technology-based solutions and implemented digital queuing systems to further reduce risk and avoid potential impolite (and unsafe) behaviour altogether^9^ (Image 3.2).

The public began to understand and adapt to new queuing norms quickly in most cases. While in some contexts physical queuing is customary (e.g. the UK), in others (e.g. Italy) ‘queuing’ in normal times may not involve an actual queue. Instead, the public may adopt alternative methods of ensuring fairness and order like taking a number or simply recognizing others who are there first waiting their turn. In those contexts, ‘queuing’ during COVID-19 may now entail physical queuing compliant with new social distancing norms. Regardless of how it was done before, queuing serves as a politeness norm to ensure fairness and orderly cooperation, but now includes considerations related to health and safety.

However, things may still go wrong even in the presence of new, clear queuing norms. In extreme examples we have seen physical altercations when people fail to observe social distancing in queues, as was the case at a Walmart store in Colorado Springs in the United States.^10^ One US resident we spoke with recalled his initial impressions when visiting a Costco warehouse store in the early weeks of the pandemic:

It was really weird because it was like two tribes. There were the people who were very courteous, who understood [the rules] and had the same fears that you did to stay away. And there were other crazy people that were just going in there, you know, that kind of a thing. And, you know, it was really tribal.^11^

Despite some of these unfortunate incidents, new politeness norms related to queuing were introduced immediately after the onset of the public health crisis. Overall, they were adopted quickly and universally in part due to their simplicity; queuing involves politeness in a physical space and entails relatively uncomplicated demands in practice.

### Dining and Drinking with Others During COVID-19

More complicated environments and diverse forms of interaction can introduce additional challenges regarding norms of politeness. For example, interaction in sites related to the food industry such as restaurants, cafes, bars, and grocery stores can add additional layers of complexity. We may need to interact with waiting staff, place an order, conduct a transaction with a cashier, or use the toilet. These sites are central to social life and therefore become places where we observe many politeness norms. We also purposefully engage with other people in these spaces; we might meet a friend for drinks after work or eat dinner at a restaurant with family. The demands and practices related to politeness norms in these spaces can vary significantly across contexts and differ by country as well.

Disruptions like COVID-19 can lead to greater uncertainty and introduce new problems related to politeness in these more complicated environments. People recognized many of these challenges to etiquette and politeness norms early on during the pandemic. Experts weighed in on how to alter the way we thought about previous social practices.^12^ Popular news outlets provided some basic advice on behaviours when restaurants reopened after mandatory shutdowns.^13^ The public had some guidance as to the new ‘dos and don’ts’ for restaurant patrons.^14^ However, different locales could adopt distinct approaches. Furthermore, each business may have a different hierarchy of concerns based on its own priorities and circumstances.

Customers face some distinct challenges in the way they engage with businesses and can become disoriented in these uncertain environments. As a customer in one place you may quickly learn some basic rules to be polite to waiting staff, while in a different place where you order your own food at the counter you might need to learn different norms regarding where and how you might wait for your order to be taken away. Restaurants have been especially hard hit and face a precarious business landscape; recognizing and accommodating these circumstances has become part of the process through which new politeness norms are being formulated. In supporting local restaurants, customers might come to terms with the fact that menus may not be complete; place orders earlier in the day in consideration of preparation and supply-chain complications; tip generously to support staff; and familiarize themselves with policies related to curbside takeaway service so that employees do not have to wait.^15^ Not showing up for a reservation has always been impolite, but the consequences for businesses in the pandemic climate are especially severe.^16^ Efforts to educate the public as to the dire financial effects could help, but some businesses may need to adopt new deposit policies so as not to rely on the politeness of their customers.

Businesses and their employees also face new challenges related to etiquette and politeness norms, with a special emphasis on obligations to ensure health and safety. Simple practices like greeting customers have to be adjusted. For example, an employee at a Melbourne restaurant described the disruption to the way they greet patrons: ‘our customers are like an extension to our family. It’s not uncommon for us to greet with a hug so this has been very difficult for us to acclimatize ourselves with’.^17^ Policies related to food preparation and service need to account for new concerns and expectations too. The seating plan for customers must now consider physical distancing requirements. Businesses should recognize new risks for viral transmission related to touching surfaces and take precautions when using specific technologies related to ordering and payment. In the United States, where it may have been customary to provide a gratuity to your waiter in cash even while paying with a credit card to ensure they keep most of the money, it may now be more polite to add the gratuity on the card to avoid touching the money, or take some precautionary steps. For example, lifestyle and etiquette expert Elaine Swann recently advised: ‘[i]f you do have to tip in cash, to put [workers] at ease, put the cash in an envelope in advance…One of the core values of etiquette is to make sure we’re doing everything we can to put others at ease’.^18^

Customers have also had to more carefully consider how they might interact with other patrons they do not know while inside restaurants. Polite behaviour when waiting to be seated and in passing other customers largely relates to physical distancing. Restaurants can help customers to be polite in this way. Normally, physical distancing in restaurants includes basic etiquette related to not touching others, respecting privacy, and being unintrusive. We generally respect a ‘personal bubble’, though the specifics can differ by country.^19^ These expectations have become especially important during the COVID-19 crisis.

Restaurants, bars, and cafes have taken measures to help patrons become aware of the need to maintain physical distancing and in many cases provided some tools to help them do it. For example, a Paris sidewalk cafe used giant teddy bears placed in seats to ensure adequate spacing between customers at neighbouring tables.^20^ Restaurants all over the world employed mannequins, dummies, and cardboard cut-outs to communicate the need for and to help enforce social distancing measures.^21^ Some locales maintained a sense of humour about how to best adhere to new public health guidelines. For example, a cafe in Germany asked patrons to wear hats with long pool flotation noodles attached to the top to help with spacing.^22^ A US bar in Maryland placed customers inside giant rolling ‘bumper tables’ to keep them two metres apart while drinking on its outdoor patio, and a restaurant in Amsterdam seated diners inside miniature greenhouses built for two.^23^

In addition to polite behaviour during interactions with other parties in a restaurant, the public has also had to adjust to new etiquette concerning eating with co-diners. For example, the way we eat differs across cultures and has changed throughout history.^24^ The COVID-19 pandemic has introduced new challenges and considerations to the way we interact with our family, friends and colleagues in environments related to food service. As a result, customers might need to change their eating habits in some contexts. For example, communal seating and sharing platters may become less common moving forward,^25^ and health risks have affected long-standing cultural traditions tied to dining behaviours.^26^ These changes could have carry-on implications. For example, research suggests that acts like sharing plates and meals facilitates cooperation and reduces competitive behaviour among acquaintances and strangers alike.^27^ Where sharing a meal was previously seen as polite, potentially building trust and strengthening social bonds, this may now prove more challenging.

### Greetings During COVID-19

Politeness norms related to other common human interactions like greetings are often situational and culturally dependent. For example, you might hug a family member or friend when you meet for a coffee, while a simple handshake would be more appropriate when meeting a colleague from work. In different cultural or professional contexts, you might demonstrate politeness with a formal bow, a rigid salute, or a casual fist-bump. We learn the best practices to facilitate smooth exchange with others through socialisation.^28^ With the onset of a pandemic, previously understood expectations regarding appropriate greetings are less clear. A polite handshake meant to initiate a pleasant social exchange might make the other party uneasy or be perceived as unsafe at best or offensive at worst. COVID-19 has turned some of the politeness norms related to greetings on their head, where an other-regarding demonstration of care may now entail abstention from previous polite behaviours. Given the health risks associated with proximity and touch, the public could benefit from a ‘hug and handshake hiatus’ and develop alternative ways to exhibit politeness when greeting others.^29^

Specific cultures and social environments have been affected in distinct ways. For example, politicians in France have urged the public to refrain from *la bise*, a customary French greeting of air kisses on the cheeks.^30^ Those living in Muslim societies face similar disruptions as a result of social distancing measures. A customary handshake followed by a hug between friends and acquaintances of the same sex has largely fallen out of practice.^31^ Disruptions and changes to the way we greet others has consequences for subsequent interactions and the trajectory of our relationships. This issue extends to the business world as well. A bank employee in Italy explained to us:

The handshake has always been considered a sign of trust in the other person, more so in the banking sector. The customer is placing their life savings in the bank’s care; this is an act of great trust. Not being able to shake hands with customers [during the pandemic] has certainly led to a situation of freezing relationships— everything is more impersonal.^32^

Likewise, when a commercial real estate agent in New York ended a lunch meeting with someone he had known for ten years without a parting hug, he remarked that it ‘felt like we didn’t close the loop’.^33^ The disruption leaves something of a ‘norm gap’ when expressions of politeness transition from one form to another. The communicative and signalling functions of civility as politeness are in flux (a point to which we will return in the next section) and we must develop alternative practices.

In more formal contexts, politicians will need to develop novel forms of official protocol to guide diplomatic exchange between leaders.^34^ How politicians interact with one another, as well as with their own citizens, has become more complicated. For example, Prime Minister Boris Johnson has had to learn how to balance conflicting politeness norms. Early in the pandemic he joked about shaking hands with coronavirus patients: ‘I’m shaking hands continuously, I was in a hospital the other night where I think there were actually a few coronavirus patients and you’ll be pleased to know I shook hands with everybody!’ He went on: ‘[a]nd I continue to shake hands’, then he smiled and concluded, ‘[o]ur judgement is wash, washing your hands is the crucial thing’,^35^ rather than abstaining from handshakes. Johnson’s statement shows how previously ingrained politeness norms around greetings might sometimes take priority for some people over new concerns for health and safety. The medical community recognizes the problematic nature of the handshake in healthcare settings,^36^ but shaking hands can represent an especially important act for politicians to relate to constituents.^37^ Johnson contracted the virus shortly after.

Public consensus regarding the potential dangers and inappropriateness of the handshake has led to a range of alternative greetings. Early substitutes included smiles and air kisses from afar, but subsequent mask requirements placed additional limits on visibility. Other gestures might include a small head incline, a hand to the heart, a namaste gesture, or even a *Star Trek*-inspired Vulcan salute.^38^ More animated greetings could include ‘jazz hands’, a footshake, an elbow bump, or a somewhat riskier fist bump in the appropriate circumstances.^39^

## Politeness Signals Going Awry

The second challenge posed by COVID-19 to civility as politeness concerns the communicative and signalling function of polite speech and behaviour. We saw in the previous chapter that ‘civility…is an essentially *communicative* form of moral conduct’ since it helps convey respect and considerateness for others.^40^ However, this is only the case if polite signals are successful and receive uptake from those at the other end of the polite interaction. If politeness signals misfire, those using them will fail to communicate their respect and considerateness to their interlocutors.

Politeness signals can go awry even in normal times. For example, people in different cultures may adopt different politeness norms regarding how to initiate a conversation; some may prefer a more direct approach whereas others may favour greater circumspection and ambiguity.^41^ Agents who are not aware of these differences and employ their ready-made vocabulary of politeness might inadvertently send the wrong signal to their interlocutors, coming across as rude, offensive, or disrespectful. This can have significant implications for the remainder of the interaction, since empirical evidence shows that the early stages of a conversation affect the extent to which the conversation will remain polite and civil in the long term.^42^ Likewise, norms of politeness concerning giving up one’s seat for older passengers on buses may be culture-dependent; while this gesture may be perceived as polite in most Western countries, it is considered rude in Japan, where older people view it as an impolite reminder of their advanced age.^43^ If you are unaware of this cultural difference, by giving up your seat to an older person you will unintentionally send the wrong politeness signal.

Politeness signals may also go awry when politeness norms are in transition or when old norms are still in place but have become contested. Take, for example, the act of a man holding a door for a woman. In the past, this would have normally been perceived as an act of politeness and chivalry, whereas in more recent times it might be interpreted as an instance of sexism by some.^44^ This may have two potential consequences. On the one hand, a man facing the choice of whether or not to hold a door for an approaching woman may make the ‘wrong’ choice and, therefore, send her the ‘wrong’ signal. When a politeness norm is in transition, it is plausible that some people still interpret it in the ‘old’ way (in this case, an act of politeness and chivalry) whereas others will assign it a new meaning (in this instance, a sexist act). Therefore, if a man decides to hold the door open but the approaching woman endorses the latter interpretation, that act of intended politeness will go awry and not have the desired effect.^45^ Alternatively, a man may decide not to hold the door open for a woman by appealing to the value of gender equality but, by doing so, may be considered impolite by the woman, if the latter still endorses traditional views concerning door-holding.^46^ Of course, holding the door for another person may also be devoid of a gender dimension and simply be intended or received as a generic act of politeness.

All these problems have been exacerbated by COVID-19. Due to the norm identification challenges illustrated in the previous section, the pandemic has made it more difficult for people to find a ready-made vocabulary of politeness to communicate their respect and consideration for others, even in the absence of cultural barriers. Since many politeness norms have become contested or are in transition, people may sometimes inadvertently come across as impolite, rude, and disrespectful: their politeness may backfire. However, this unintentional impoliteness may result not only from the use of the ‘wrong’ politeness signals but also from the decision not to use *any* politeness signals, given the uncertainty surrounding politeness norms and practices. For example, if we are no longer sure whether to greet others with a handshake, an elbow, or in some other way, we may decide not to greet them *at all*. But those we interact with may interpret abstention itself as rude and impolite.

In the remainder of this section, we consider three examples of how politeness signals can go awry within the context of the current pandemic. We focus specifically on workplace communication (both online and face-to-face), mask wearing, and interactions in everyday spaces such as sidewalks, gyms, and outdoor exercising areas.

### Workplace Communication During COVID-19

COVID-19 has created new points of confusion as to norms of communication in the workplace, especially regarding the best ways to express politeness in email correspondence and proper etiquette during meetings. The way we communicate politely via email may include elements related to aesthetics, length and content, as well as greetings and other social niceties.^47^ A strict adherence to decorum in more formal correspondence might signal an appropriate level of professionalism and regard for the recipient. Employees who frequently use email also recognize that some transgressions and impolite ‘flaming’ behaviours (e.g. all-caps that might be perceived as aggressive) can have adverse effects, creating tensions, antagonistic relationships, or overt conflict in the workplace.^48^ Those should be avoided.

Making adjustments to our email correspondence during the pandemic entails mostly only minor accommodations to still effectively signal politeness. However, it is crucial that we demonstrate an awareness that we are all living through challenging times. It is polite to acknowledge the broader disruption to our social lives, as well as how we relate to others in workplace roles.^49^ For example, leading off a correspondence with ‘I hope that this email finds you well’ may sometimes feel inadequate. Likewise, as we conclude our emails, many of the old sign-offs like ‘all the best’ or ‘regards’, may no longer suffice. These expressions seem to fall short in signalling that you are a good colleague. Effectively signalling politeness in correspondence during COVID-19 requires an even greater degree of compassion and genuine concern for the other party.^50^ Employees ultimately need to transform workplace communication into something of a ‘recognition sandwich’, acknowledging the situation and the well-being of others at the beginning and end of the correspondence.^51^ This can prove more difficult in some contexts than others. For example, the authors found it quite challenging to encourage colleagues to ‘have a good weekend’ at the end of an email when the entirety of Melbourne, Australia lived through strict lockdown measures that included a curfew and only one hour outside the home for exercise or groceries, all within five kilometres of their homes.

The content of email correspondence also has implications for the signalling function of politeness. In the context of a global pandemic, making simple everyday requests of others that may have previously been quite normal can now seem unnecessary, insensitive, or impolite given other concerns and responsibilities. For example, academics gently nudging a colleague about a submission deadline for a book chapter or peer-review comments on an article manuscript could come across as impolite. However, scholars in these positions still need reviews and chapter submissions as part of their jobs. In light of the new circumstances, people might employ new work-around practices of politeness; perhaps recognising the plight of others, pointing out the apparent absurdity of the request given the state of the world, or even using humour in a strategically self-deprecating way to acknowledge what may be an inappropriate request. These practices conceal a business-as-usual outlook in some way and constitute a new way to be polite, send the ‘right’ signal, and reduce incidents of perceived inconsiderate behaviour. Our ready-made norms of politeness may not be enough, and in this context we need to add an extra step. This might include an additional performative layer to ease what might be an objectively inappropriate request as the other party could be bereaved, suffering from acute mental stress from long-term social isolation, or be overly burdened with increased caring responsibilities, in addition to their everyday work requirements.

Expectations regarding interpersonal interactions during work meetings are also less clear during the pandemic. Public health measures have forced employees in many industries to work from home. Many meetings now take place remotely, using popular video conferencing tools like Zoom. Employees face uncertainty and new challenges regarding signalling politeness and respect in virtual environments. The norms regarding best practices and behaviour in these spaces are still emerging. Zoom has provided its own guidance regarding video meeting etiquette with some simple suggestions like introducing other participants, keeping a professional visual background, and looking into the camera when you speak.^52^ Popular media commentary has helped to identify particular social *faux pas* to avoid and best practices to ensure a more positive experience.^53^ There is an emerging consensus about what may be deemed polite or impolite. Some key issues include muting your microphone when not speaking, or your video when performing certain actions that might be perceived as visually unpleasant by others (e.g. eating); minimizing background noise; being mindful of camera position and stability; avoiding multitasking like writing emails or completing other work tasks; and remaining mindful of your appearance and dress.^54^ Users should also consider potential technological issues like a weak Internet connection and be prepared to engage without the video function in those cases. However, turning off the video has now come to be seen as impolite by some, as an indication that the user might not be fully engaged with the conversation or meeting, and simultaneously focused on other tasks.

### Politeness and Mask Wearing During COVID-19

COVID-19 has high transmission rates and the virus can spread through surface contact and via airborne particles. Mask wearing has therefore become an important preventative measure to reduce its diffusion and mitigate health risks now associated with so many activities in public life. However, a mask can hinder our ability to communicate with others, especially people with hearing difficulties and those who speak other languages. Wearing or not wearing a mask is also a signal unto itself. Beyond its functional role, a mask can communicate messages to other people about yourself and how you see others. When masks are not required by law, wearing one might send ambiguous signals. While some could see it as an expression of other-regarding behaviour, others could interpret it as a sign of distrust. For example, a dentist in Australia reported having been subjected to verbal harassment when a customer yelled at her for wearing a facemask in a supermarket at the onset of the pandemic. The Australian public had not yet come to see wearing a mask as a demonstration of other-regarding behaviour and the customer was offended not being able to see the dentist’s face. In reality the dentist wore the mask to keep others safe because of the high level of contact with others in her profession.^55^

The type of mask can complicate signals even further, and the meaning and intention behind mask wearing may not always be clear. A large face shield, for example, might be seen as excessive and antisocial, while a simple cloth covering, although less effective at preventing transmission, could be seen as a gesture of solidarity given the need to ration medical grade masks.^56^ Furthermore, *refusing* to wear a mask can signal dissent and communicate a specific political position, representing a disregard for public safety and an empty act of defiance to some audiences.^57^ Conversely, in polarized political climates, wearing a mask could be seen as a ‘smug liberal’ act by some.^58^ However, a mask-wearing culture can change quickly and its meaning can transform just as fast.^59^

Wearing a mask may also obscure other ways of sending signals. Smiling, frowning, and other facial expressions help to signal emotions like happiness, surprise, anger, or disapproval, which are often crucial to polite interactions.^60^ However, different parts of the face are responsible for communicating emotions. Positive emotions generally emanate from the bottom half of the face and widespread mask-wearing provisions keep them hidden, while negative emotions are more visible through the top half of the face. A reduced capacity to visibly communicate emotions or perceive others’ expressions inhibits the signalling function of polite facial expressions.^61^ For example, one woman in Italy reflected on this challenge:

We Italians used to greet relatives, friends, but also acquaintances with a handshake of course, but also with hugs and kisses. I haven’t seen this done since [the pandemic began]. Now we greet each other with a ‘hello’, perhaps raising our voice because the mask limits a lot. It happened to me personally running into an acquaintance, instead of a hello I greeted her with a simple smile. Then I realized that since I was wearing both a mask and sunglasses, she must have thought that I had not responded to her greeting and I felt guilty.^62^

We might develop alternative ways to overcome these new signalling limitations that masks create. For example, as one potential solution to the communication difficulties that result from reduced facial visibility, some manufacturers have started to create transparent ‘civility masks’ with a new ‘smile-through’ design.^63^ Alternatively, we may need to place more emphasis on the importance of tone and use more body language, given the potential impediments that wearing a mask poses for facial expressions. Our words and gestures, along with the manner in which they are delivered, can be important (im)politeness signals. For example, sitting with arms and legs crossed may signal closure, while facing away or looking in a different direction from someone with whom you are engaged in conversation may communicate discomfort or disinterest. Carefully choosing our words and gestures will become crucial during the pandemic.^64^ A mask might also force people into uncertain situations where there might be a tension between different politeness norms. In these cases, people may need to make judgments about the signals they want to send and assess whether the other party has received the intended message. For example, when crossing paths with a friendly neighbour, showing your face and trying to be kind and social, even from a distance, may actually come across as inadvertently impolite during the pandemic.

Another solution that many people have adopted in response to mandatory mask wearing involves the use of personalized homemade face coverings, which were initially a pragmatic response to mask shortages among medical workers. Personalized masks also have the potential to communicate a range of messages^65^ and provide new opportunities to signal politeness to others. The form and design can allow its wearer to express various messages. Pattern and image content might provide a space to communicate politeness, as well as identity, personal affinities, or a humorous message to others. Furthermore, some designs can be more inclusive or message-neutral than others, such as Hawaiian aloha print designs.^66^ People might also be able to use personalized masks to signal a political identity that expresses solidarity with the LGBTIQ+ community or the Black Lives Matter movement through specific colour patterns or messaging.^67^ Alternatively, masks could include aggressive, offensive, or highly politicized content,^68^ and some mask wearers might signal an oppositional stance in the way they wear a mask incorrectly, or alter it to visibly counteract its intended public health function.

### Politeness in Everyday Spaces During COVID-19

COVID-19 has disrupted etiquette and politeness norms in contexts where they were previously well-established. Simple gestures like holding a door open for strangers or delaying a lift to allow for another passenger are no longer straightforward acts of politeness that signal social cooperation and consideration. People have had to adjust previously polite behaviours to accommodate new priorities that place health and safety first. Holding the door for a stranger would unnecessarily place you within the recommended physical distancing guidelines. However, you might still signal the intention of being polite by communicating why you refrained from holding the door. One etiquette and culture expert explained: ‘Kindly tell them, “Normally I’d hold the door for you, but I’d be within 6 feet”’.^69^ The public is still learning to navigate new health risks and how to share spaces with others in the context of the pandemic.

Norms of politeness are not static. Even prior to COVID-19 we lived in a world characterized by uncertainty regarding how to be polite in different contexts and environments, and the public grapples with contested norms or norms in transition on a regular basis. The practice of holding doors, already mentioned at various points in this chapter, has its own complicated history as an everyday ritual that may differ based on factors such as age, gender, or social status, among others.^70^ Likewise, expectations around politeness in online discussion groups are still evolving.^71^ Tone and directness of speech during in-person conversations can be seen as impolite to varying degrees across time and context, but individuals can make accommodations with relative ease.^72^ The global pandemic has disrupted (or further disrupted) previous politeness norms in everyday spaces and the public will need to adapt and learn to be polite together.^73^ This may require novel guidance in line with new knowledge about risks related to viral transmission, but also general pragmatism on the part of the public at large. Politeness norms may also evolve in ways that depart from health and business guidelines. For example, new lift guidelines at an Australian public university campus suggest that two people are allowed inside at the same time based on social distancing guidelines. Two passengers can fit in the space and still maintain a distance of 1.5 metres. However, the authors found that the polite gesture in practice quickly became for the second waiting passenger to allow the first to use the lift solo and wait for the next one. In this kind of situation, the passenger might adopt new practices like pressing the ground floor button as they exit the lift to send it back down immediately and to reduce waiting time below.

Another example of uncertainty regarding norms of politeness in everyday spaces involves our behaviour on the street. Social distancing norms have taken on a greater importance during the course of the pandemic. By the middle of 2020, maintaining a greater degree of physical distance from others became the best practice in preventing community transmission. Overt efforts to adhere to distancing guidelines became the most important way to signal politeness. However, before the COVID-19 crisis was declared a global pandemic on 11 March, many people still made decisions on how to behave in relation to others on the street in relation to other politeness norms. Prejudice towards people of Chinese or East Asian descent became more pronounced in some places owing to the origins of the virus. An academic from Israel described his own experience regarding uncertainty on how to behave politely in the street:

The other day I was walking my dog; it was very early in the morning and the sidewalks were empty. Then suddenly I noticed that opposite me on the sidewalk was a Chinese person walking towards me. I suddenly felt that I was afraid to pass by him, and I thought this was terribly strange. I kind of forced myself to continue walking towards him and not be afraid, as it would appear very impolite and maybe humiliating to move to the sidewalk on the other side of the street.^74^

This shows the academic’s difficulty in choosing the correct action in terms of his own safety, but also his awareness of the potential risk of communicating distrust and fear by crossing to the other side of the road. At that moment, crossing the street might have been perceived as an act of prejudice, but perhaps not doing so could have been seen as a disregard for the other person’s safety in light of the emerging threat presented by the virus. As the pandemic progressed, the public began to perceive efforts to create additional physical distance (including crossing the street, if necessary) as the polite thing to do.

**Image 3.3:**
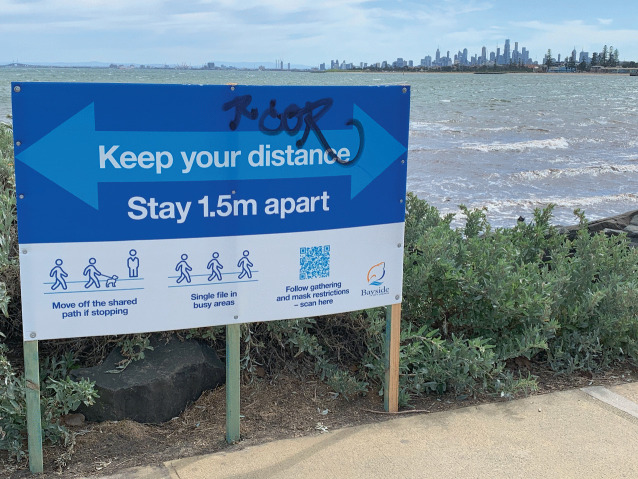
Guidance for walking path rules including distancing, stopping, and behaviour in busy areas

**Image 3.4:**
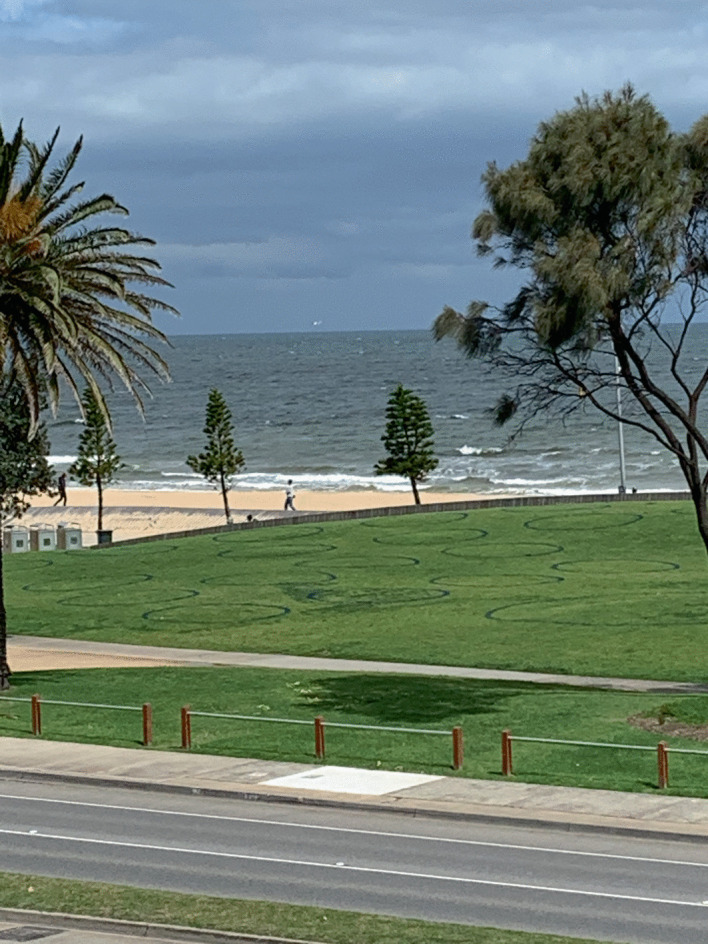
Circles on the grass to help people observe social distancing rules

Many places around the world have experienced restrictive measures aimed at curtailing the spread of COVID-19. For example, between August and September 2020 residents in Melbourne, Australia became subject to a Stage 4 lockdown that included a stay-at-home mandate that only allowed for travel to buy groceries and one hour of exercise within five kilometres of one’s homes, accompanied by an 8pm–5am curfew. Many people began to take regular walks outdoors and were not prepared to behave appropriately and exhibit politeness to others in these spaces. A simple acknowledgement of other people in passing would no longer suffice. Given the new demands around physical distancing, people needed additional guidance on how to behave politely on walking paths along the bay, in public parks, or when using outdoor exercise equipment in proximity to others (Images 3.3 and 3.4).

**Image 3.5:**
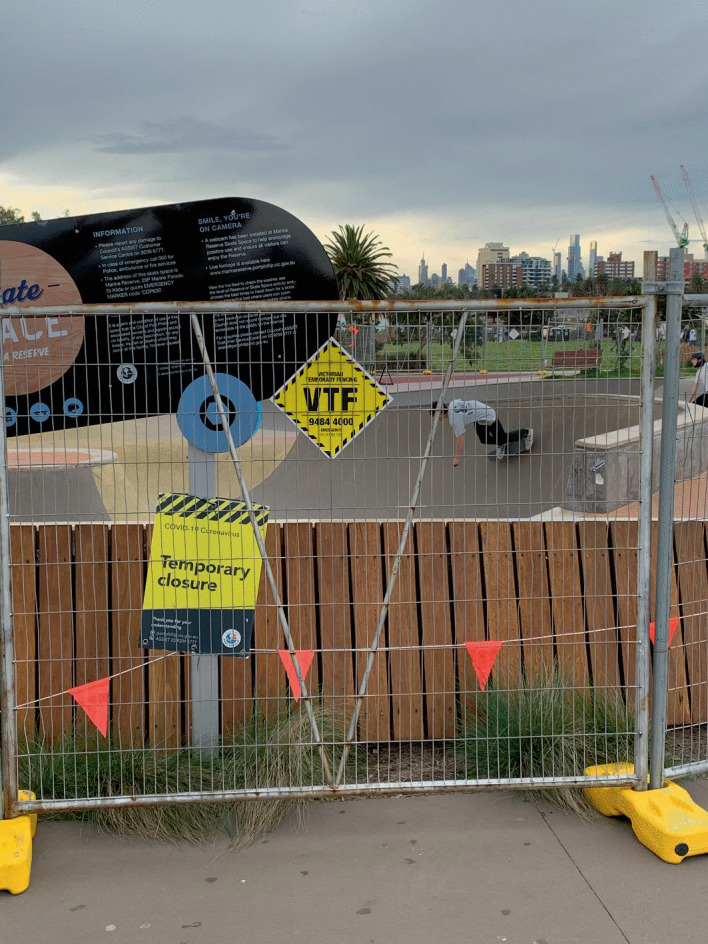
A skater failing to comply with COVID-19 rules

The state government restricted access to some spaces at various times during the lockdown, including skate parks and outdoor gym equipment. Some residents chose to comply and others ignored these guidelines. When the use of outdoor fitness spaces became permissible again in Melbourne, users had to adapt to new politeness norms. Previously, the dominant way to signal respect and cooperative behaviour involved simple, considerate acts like time limitations on pieces of equipment or acts of fairness like waiting turns. However, politeness now requires greater consideration, with general gym etiquette emphasising norms specific to hygiene and cleanliness, and providing enough space between yourself and other users. You should also wear proper attire, use a towel, wipe down equipment after use, and refrain from excessive socialising.^75^ If you decide to perform yoga or conduct a fitness class in a public space, be mindful of how your behaviour might affect others’ ability to share the space. Residents should also remain mindful of official policies and comply with public safety measures (Image 3.5).

In this section we have so far examined unintentional and involuntary acts of impoliteness. However, given its signalling function, people can also be *intentionally* impolite. While they might sometimes do so in order to express disagreement about specific norms (without challenging the need for ‘a’ norm), or to demonstrate dissent from unjust government policies,^76^ in many cases they might simply be acting out of self-interest and showing a general disregard for others. We might counter the latter type of intentional impoliteness in several ways, by introducing avenues and pressures toward compliance while also constructing barriers to non-compliance. This can be achieved through interventions around costs and by introducing mechanisms for informal or formal sanction for violating norms. Programming that helps foster awareness, legitimacy, and acceptance of particular norms can also help encourage compliance. When, instead, impoliteness emerges from dissent, governments might need to prioritize the creation of spaces and tools for channelling that dissent into engagement, debate, consultation, and deliberation, so as to reduce the need for purposeful acts of impoliteness that may lead to greater social tensions in society rather than cooperation.

## Impoliteness and New Barriers to Social Cooperation

As we highlighted in the previous chapter, one of the key functions of civility as politeness is to act as a social ‘lubricant’^77^ by facilitating social interaction and cooperation and reducing the potential for social tensions.^78^ Uncertainty regarding politeness norms and their signalling function (as discussed in the previous sections), combined with general anxiety resulting from COVID-19, can create a barrier to social cooperation. It is important to recognize that impediments to social cooperation can be either intentional or unintentional. On the one hand, a person might decide to deliberately breach norms of politeness, or use the ‘wrong’ ones (given the circumstances) in order to signal their disrespect for others and thus generate social tensions. On the other hand, an inadvertent impolite action might have a similar negative effect on social relations.

For the first scenario, imagine a man who holds the door for female colleagues at his workplace daily when he *knows* the action will be perceived as sexist and disrespectful (e.g. because those colleagues and/or his manager explained this to him before). Or in the political sphere, consider when US President Donald Trump refused to shake hands with German Chancellor Angela Merkel during a visit to the White House in 2017 despite requests from journalists and from Merkel herself. Trump’s behaviour was interpreted by some as ‘a sign of the tensions between the two leaders’,^79^ a tension which, one could argue, Trump deliberately sought to create. The intentional refusal to shake hands can be inferred from contextual cues. First, Trump is usually keen to shake hands with other political leaders, as exemplified by his participation in a traditional ‘group handshake’ at an Association of Southeast Asian Nations (ASEAN) summit in Manila in November 2017,^80^ or when he shook Japanese Prime Minister Shinzo Abe’s hand for an impressive 19 seconds in February 2017.^81^ Abstaining from a handshake with Merkel could be seen as a clear departure from a norm of politeness Trump normally complies with enthusiastically.^82^ Second, as one CNN journalist pointed out at the time, ‘[t]he tense moment between the American and German leaders [came] after Trump repeatedly bashed Merkel on the campaign trail and accused her of “ruining Germany”, citing the nation’s policies allowing refugees into the nation’.^83^ In other words, the handshake refusal could be understood not as an isolated incident but rather as part of a broader tense relationship between the two leaders at the time.^84^

As an instance of *unintentional* impoliteness leading to social tension, consider another incident involving Trump where he walked in front of Queen Elizabeth II during his royal visit to the UK in July 2018.^85^ This breach of protocol generated significant interest and immediate negative reactions on social media, even among those who considered themselves critics of the British monarchy. For example, one Twitter user said: ‘I’m not a monarchist by any stretch of the imagination but this is such an insult to Britain. Absolutely clueless, classless, thoughtless, lacking in any dignity and without a shred of respect’. Another wrote: ‘[t]his is an important point. I see a lot of people bashing the monarchy, and okay. Whatever. She’s a 92 year-old woman that he’s walking with. Simple respect and decorum would cause him to walk at her pace regardless of who she is’. A third one stated: ‘[m]e: I hate the Royals, they are leeches on society. Also me: DONALD TRUMP DON’T DO THAT TO THE QUEEN’.^86^ These reactions clearly show that breaches of protocol, decorum, and politeness norms can create social tensions, in this case between two countries and their citizens. Tensions also arose between the UK and Italy when then Italian Prime Minister Silvio Berlusconi shouted in the Queen’s presence during a G20 summit photo-shoot at Buckingham Palace in April 2009.^87^

Social tensions resulting from breaches of politeness norms may also arise outside of political contexts. They have the potential to undermine business relationships, especially in multicultural business contexts where politeness norms grounded in different cultural and religious traditions exist. For example, when conducting business in the United Arab Emirates (UAE), ‘one never extends their left hand and one does not address another with their first name unless having been given permission do so’, and ‘[i]t is advisable to ask open ended questions that will not require a yes or no answer. Furthermore, it is important to “save face” with all communication conducted in a harmonious and non-aggressive style’.^88^ Failing to comply with these and other related politeness and etiquette norms is likely to cause tension and undermine business relationships.

Road etiquette provides another area to illustrate the relationship between politeness norms and the easing of social tensions. Of course, every country has legal rules regulating traffic on the road. However, these may not always be sufficient to guarantee peaceful and smooth interactions among drivers. Road accidents can escalate from tensions on the road, more often a result of a drivers’ temperament and lack of politeness than infringements of traffic laws.^89^ Conversely, not cutting other drivers off or giving them right-of-way are acts of politeness that can contribute to easing social tensions on the road and thus reduce road rage and the risk of accidents.^90^ Likewise, using a ‘friendly wave’ to thank other drivers when they give way or pull over and let you pass (even when this is legally required) can go a long way towards creating a more peaceful (and, therefore, safer) road experience for all drivers.^91^

Within the context of the COVID-19 pandemic, uncertainty concerning norms of politeness has created additional moments for social tension and new barriers to cooperation. As we have already pointed out, new politeness norms emerged soon after the initial onset in some areas. However, ongoing uncertainty in many contexts may lead people to act impolitely in ways that risk undermining smooth social interactions and cooperation. In some cases, impolite action may be driven by misguided beliefs, as when people hoard goods from supermarkets, shouting at and pushing one another in the unfounded fear of shortages. In other cases, people may intentionally and consciously act impolitely for self-interested reasons, because they do not want to spend the time required to learn about or observe certain politeness norms (e.g. when they decide not to clean shared gym or outdoor exercise equipment after working out). In the following subsections, we engage with three examples that show how impolite behaviour during COVID-19 can undermine social cooperation: snitching, politeness in the workplace, and the impact of (im)politeness on international cooperation.

### Snitching During COVID-19

Snitching is the act of informing on another. There are, of course, moral implications related to snitching, and there is a growing debate on the related topic of whistleblowing in political theory and philosophy.^92^ Etiquette related to snitching is endowed with its own social norms and expectations in how it is seen and practiced. As the famous saying ‘snitches get stitches’ suggests, snitching is often understood as something that ‘you are not supposed to do’, and which may result in social stigma and ostracism on the part of others, or even worse.^93^ All of this suggests that in addition to being a morally significant behaviour (the permissibility of which may be contested), snitching is also relevant to the issue of politeness. More specifically, snitching is impolite since it contravenes norms of social etiquette which demand that we abstain from snitching on others. And, indeed, like most impolite acts, snitching can have negative implications for social cooperation and increase social tension.

Snitching has become a prominent issue during the COVID-19 pandemic. In the context of stay-at-home orders and laws around lockdown measures, some people have been reporting fellow citizens, neighbours, and businesses that violate the rules. However, many of those reporting on others have become targets of public shaming and criticism, as when in July 2020 someone in Melbourne posted a photo of and criticized people in a park not wearing masks, and was strongly condemned for doing so.^94^ Likewise, when a Melbourne pizzeria received an order for eight pizzas, and its owner posted comments suggesting that perhaps he had the duty to report this to the police (since a party of eight people contravened social distancing rules at the time), some of the responses he received included the following: ‘I would mind my own business and not make assumptions’ or ‘[b]e grateful for the business and don't just assume’.^95^ While in both examples there were those who supported snitching, the overall response suggests that the act of snitching is often frowned upon and socially stigmatized.

One Melbourne resident we spoke to recalled hearing neighbours throwing a party in violation of lockdown rules. Given the potential public health and safety effects, one might understand snitching in this context. However, she ultimately decided to adhere to the general ‘abstention from snitching’ politeness norm, and explained:

I am hesitant to get police involved when I do not know the full circumstances and there is not an immediate danger: I wasn’t completely sure where the party noise was coming from—there were at the time two separate residences that had people over—and there may have been a reason for people to meet, such as shared custody arrangements. Some of my friends had also already mentioned to me that police wouldn’t attend for gatherings of less than 10 or 20 people, so by contrast the potential numbers at the neighbours’ gatherings were lower and it would have been a waste of my time to notify the police. In discussing the situation with my partner I reflected on how I was feeling about missing significant moments in our lives with family and friends, and how other friends of mine were experiencing the same thing. So we just closed the patio doors and tried to make the best of things together.^96^

Violating an anti-snitching politeness norm can have negative implications for social cooperation. Some have argued for example that ‘hotlines for reporting “non-compliance” [are]…evil tools that will damage the very trust we’ll need to rebound from this crisis’.^97^ Likewise, political philosopher Daniel Weinstock has pointed out that ‘[i]nforming against our neighbours is highly corrosive to social relations, in ways which may continue to have echoes after this crisis is over’, and that it may often be preferable to maintain a ‘certain amount of good faith among our neighbours’.^98^ Clearly, then, snitching during the current pandemic risks undermining social cooperation. Some have referred specifically to ‘an etiquette to dealing with someone flouting rules that defy basic social behaviour’.^99^ Rather than snitching, this etiquette requires that we step up and challenge our fellow citizens and neighbours directly, when necessary. As suggested by etiquette and protocol consultant Nancy Kosik, when doing so,

you should be polite, assertive and ensure your body language is friendly. Don’t yell from your car. Maintain an appropriate distance, and maintain a positive tone…You should greet the group politely…and then say something like, ‘I don’t know if you've heard, but the government is urging us to speak up if we see people putting themselves at risk. I’m asking you to please wrap up and go home for obvious reasons, even if you’re feeling fine’…Once you’ve said your piece…stay there. ‘Don’t just leave. See them disassemble their group. Reinforce that you mean what you say’.^100^

Kosik’s recommendation signals the importance of politeness on two levels. First, challenging others directly, rather than snitching on them, is the polite thing to do. Second, the challenging itself should be done in a way that is polite, lest it be ineffective, counterproductive, and escalatory.

On the latter point, famous etiquette expert Judith Martin, aka Miss Manners, provides similar advice. When facing those who fail to wear masks or comply with social distancing norms, she argues,

[y]ou don’t insult them because, among other things, it doesn’t work…When you start screaming at people, ‘You’re trying to kill me’, and, ‘Back off’, and that sort of thing, do they say, ‘Oh, excuse me’, and back off? No, they get hostile…[therefore we should instead say]… ‘I think we should have more distance here’…You're giving the person the opportunity to do what you want that person to do without being embarrassed…because if they get embarrassed, they’re going to get mean’.^101^

Once again, we can see the clear connection between politeness and social cooperation. Even if we avoid snitching and decide to directly challenge those who violate COVID-19 rules, we should do so politely, otherwise we risk increasing social tension.

### Workplace Politeness During COVID-19

The workplace provides another example concerning the potential negative effects of impoliteness on social cooperation during COVID-19, especially around relationships between co-workers. COVID-19 has clearly increased the level of anxiety and paranoia within the workplace, with colleagues becoming suspicious of one another for a variety of reasons and thus increasing social tension. For instance, a company spokesperson of UK-based health and safety software providers Protecting.co.uk stated:

I’ve heard many workplace horror stories since the coronavirus outbreak, of rumours being spread and increasing tensions between colleagues…People are stifling coughs to avoid bullying and harassment from colleagues, while others are feeling like they’re being avoided unnecessarily because of office hearsay.^102^

In a specific case, a worker who became the target of suspicion recalled:

I’ve been allergic to the air freshener in the office for the last five years, but now everyone seems to be on high alert and has a huge problem with me when I sneeze. I know we have to socially distance from each other, but they are going out of their way to avoid being anywhere near me. I find it really rude, and I’m actually quite upset.^103^

Likewise, especially at the early stages of pandemic, when domestic and international travel was still allowed in many countries, there were cases of employees returning from overseas trips becoming the target of suspicion, accusations, and social stigma. Both authors returned to Australia from overseas business travel in late February and early March 2020 and experienced moments of minor apprehension and uncomfortable humour related to potential exposure to the virus during travel. All these examples show that COVID-19 can result in the disruption of norms of politeness and courtesy in the workplace, thus undermining collegiality and workplace cohesion.

The disruption of norms of politeness in the workplace concerns not only relationships between co-workers, but also between workers and customers. This has been especially pronounced for businesses with public-facing staff, such as supermarkets and other kinds of retail stores. There have been many cases where tensions between staff and customers escalated due to the latter’s rude behaviour, thus undermining the social cooperation that is central to everyday commercial transactions within the marketplace. For example, a bank employee in Italy described interactions with customers during the first wave of COVID-19:

Several quarrels and disagreements with customers have arisen due to the latter’s noncompliance with the rules…Many customers enter the branch without a mask, others do not want to wait outside for more than a few minutes and become hostile towards employees…There have been multiple attacks on bank employees—stones have been thrown at some branches…For us bankers it hasn’t exactly been a good time, we were at the forefront in the days when there were thousands of deaths.^104^

Kind and polite behaviour on the part of both staff and customers, e.g. via common courtesy expressions such as ‘please’ and ‘thank you’, can indeed go a long way towards facilitating smooth and efficient interactions and transactions. Disruptions to or noncompliance with these norms of politeness can result in closure and may undermine the smooth working of the marketplace. During the early stages of the pandemic in Australia, for example, many supermarket workers became the target of rude and abusive customers and refused to serve them. Furthermore, the presence of impolite customers led others to leave the stores.^105^ Both responses to this impolite behaviour were clearly deleterious to smooth and efficient market transactions, by depriving some customers of staff assistance and some stores of customers. In response to these incidents, Barbara Nebart, Newcastle branch secretary of the Shop, Distributive and Allied Employees’ Association (SDA), stated:

[P]lease respect our retail workers…They are not supplying the goods. They are just trying to serve you to the best of their ability…If companies have put limits on buying items, please respect those limits…I think people just need to calm down. Everyone should try and be a little kinder to each other…I must say, so far companies are trying to do the right thing. Woolworths [supermarkets] have started twice hourly announcements to please respect staff.^106^

The fact that supermarkets had to proactively make announcements to ask customers to be polite towards their staff not only signals the seriousness of the problem, but also the importance of educating people to politeness. Politeness, we explained in the previous chapter, involves an agential dimension. We must make an effort to acquire information about norms of politeness, context, and other factors on which our polite behaviour depends. But often we cannot do this on our own, and we need others’ support. Politeness is often built together, in cooperation with others. In this example Nebart and Woolworths tried to do just that: explaining to customers the sources of the problem, in order to mitigate their anger and frustration, and inviting them to be kinder towards supermarket staff. Hopefully these kinds of interventions can help to recover politeness and the resulting social cooperation.

Incidents involving rude behaviour have affected not only banks and supermarkets but also other businesses. For example, in July 2020 one woman in San Francisco allegedly decided to urinate on the floor of a mobile phone store to show her opposition to its policies regarding mandatory mask wearing.^107^ This incident shows yet another extreme violation of norms of politeness regulating and facilitating interactions between staff and customers. But it also suggests that COVID-19 might have created greater uncertainty as to how to communicate objections to new norms and social expectations—how best to express disagreement and exercise noncompliance. If incivility (as impoliteness) can be used to signal dissent,^108^ those who take action might not always be able to rely on a ready-made vocabulary of speech and behaviour: sometimes, people may need to be creative in order to be uncivil and communicate their dissent.

Tensions between staff and customers in the airline industry have also become especially strained during the pandemic. Travel, as we have already stressed, has been severely disrupted by COVID-19. However, some airlines have continued to operate, albeit often only via domestic routes and with reduced frequency. During one incident in the US, tensions erupted on a flight from Denver to Los Angeles when a couple refused to wear masks despite repeated requests from flight attendants. They invoked their ‘constitutional right’ and as a result the flight was delayed until the couple was asked to disembark. The delay caused disruptions for the other passengers onboard, some of whom feared they might miss their connecting flights. One passenger remarked: ‘I can’t believe that you were so rude. Why? Why couldn’t you just wear a mask?’.^109^ This example shows how impolite behaviour can clearly undermine social cooperation in such everyday situations as travelling and catching a flight. Even when there are clear policies regulating people’s behaviour (in this case, the airline had a no-exception mask policy), full compliance cannot be achieved solely via formal means. To be more precise, full compliance *can* be ensured via formal enforcement mechanisms like insisting that the couple disembark from the aircraft. Likewise, another US carrier started to issue ‘yellow cards’ to passengers who refuse to wear masks on its flights, explaining that this might lead to bans from future flights.^110^ However, these more formal sanctions may not always be conducive to (or sufficient for) greater social cooperation. If every traveller behaved like the couple in the example, social cooperation would be undermined or at least seriously disrupted. Polite behaviour is the lubricant of social cooperation because it helps facilitate interactions without recourse to (or alongside) more formal sanctions and regulations.

### Politeness and International Cooperation During COVID-19

A final example in this section concerns politeness and international cooperation. For instance, in May 2020 Australia called for an independent international inquiry into the origins of the COVID-19 pandemic in China. The request added additional stress to ongoing tensions between the two countries, with terms such as ‘downright despicable’, ‘petty tricks’, ‘menacing’, and ‘irrational’^111^ being used by various diplomats and MPs. In response to Australia’s move, China’s ambassador to Australia Cheng Jingye said:

The Chinese public is frustrated, dismayed and disappointed with what you are doing now…if the mood is going from bad to worse, people would think why we should go to such a country while it’s not so friendly to China…The tourists may have second thoughts. Maybe the parents of the students would also think whether this place, which they find is not so friendly, even hostile, is the best place to send their kids to. So it’s up to the public, the people to decide. And also, maybe the ordinary people will think why they should drink Australian wine or eat Australian beef?^112^

Some have tried to justify this kind of approach. For example, Ruan Zongze, vice-president of the China Institute of International Studies, stated:

Calling for an investigation is just buck-passing behaviour…[this is just]…self-defence against irresponsible accusations made by Western political figures. China isn’t provoking anything…China should choose to be tough on people who just try to throw mud at our country, because they never hesitate to demonize and degrade China. How can you be polite to impolite people?^113^

Therefore, China’s own impolite behaviour towards Western countries is viewed here as a response to those countries’ earlier impolite acts, manifested in demands for an international investigation in the origins of the global pandemic. But this can only contribute to a spiral of impoliteness that risks undermining international cooperation. The Australian Government perceived the aforementioned statement by China’s ambassador as a threat. And verbal threats are certainly a clear example of impoliteness,^114^ especially in a context like international relations and diplomacy: relationships between states may become difficult when one or more of them adopts this kind of language. Indeed, Australia’s foreign minister responded to the Chinese statement in the following way: ‘[w]e reject any suggestion that economic coercion is an appropriate response to a call for such an assessment, when what we need is global cooperation’.^115^

One point to stress is that the spiral of impoliteness in this example also involves references to international students, who have been particularly affected by COVID-19. Soon after the onset of the pandemic, many countries around the world closed their borders to international students by imposing various travel bans. For example, in March 2020 Canada issued new statements and policies intending to prohibit entry by new international students and only allowing those who previously held a visa.^116^ Likewise, Australia closed its borders to international students in March 2020,^117^ having previously banned all travel from mainland China at the beginning of February 2020,^118^ a measure considered controversial by many at the time. More generally, international students, especially those from China, have become targets of offensive and hostile comments in a number of countries, leading many to feel unwelcome as a result.^119^ Even when this has not escalated into incidents of racism and hate speech (discussed in the next chapter), it has created a climate that could impede international cooperation. This is exactly what emerges from Cheng Jingye’s aforementioned statement that ‘[m]aybe the parents of the students would also think whether this place, which they find is not so friendly, even hostile, is the best place to send their kids to’.^120^ This student-boycott threat could be seen as impolite and is in itself a response to a broader impolite and hostile environment. An exchange of this kind can have serious negative consequences for international cooperation since international students can play a significant role as cultural mediators contributing to smooth international relations.^121^

In another example, consider US President Donald Trump and other politicians’ use of the terms ‘Chinese virus’ and the ‘Wuhan virus’ to refer to COVID-19. In addition to causing allegations of racism, the use of these terms had the more immediate effect of preventing G7 countries from agreeing on a joint declaration at a meeting in March 2020. It constituted something of a breach of diplomatic decorum. According to a diplomat from a European country, ‘[w]hat the State Department has suggested is a red line…You cannot agree with this branding of this virus and trying to communicate this’.^122^ Clearly, in this case, the use of inappropriate language by the US administration prevented or delayed international cooperation to tackle COVID-19: if countries could not even agree on how to refer to COVID-19, it is difficult to see how they could agree on a common strategy for tackling it. Furthermore, the branding itself may also already contain certain normative assumptions regarding the policy response required to tackle it. For example, calling COVID-19 the ‘Chinese virus’ may signal not only one’s views about where the virus originated but also about who is mainly responsible for its origins and spread (i.e. China) and, therefore, what should be done about it (e.g. asking China for reparations).^123^

To minimize disruptions to international cooperation in cases like the one concerning Australia and China, governments should recognize the consequences that impolite statements and behaviour have on international relations. Countries make decisions based not only on material interests and norms, but also on their perceived treatment and status recognition by other states.^124^ Impolite and disrespectful behaviour by officials can harm cooperation and stoke tensions. Each country should take steps to renew its commitment to diplomatic decorum and exercise restraint in criticism and threats of economic retaliation. These actions, and any dispute settlement, should take place through existing international institutions that are designed to reduce conflict and strengthen cooperation.^125^ Moreover, each state might look for alternative ways to repair deteriorating relations and challenges to social cooperation. For example, Australia and China might work to improve regional agreements and trade partnerships to mitigate the potential breakdown of social cooperation caused by these impolite exchanges.^126^

Besides the broader geopolitical implications of impoliteness, there are also more specific issues faced by politicians and diplomats at the micro level, as they engage with each other in meetings and other diplomatic contexts. One of the main consequences of COVID-19 has been the transition of many international and diplomatic interactions to the online sphere. This has two main implications. First, social distancing has removed many of the opportunities for physical interactions and displays like a diplomatic handshake. These types of actions demonstrate that one is a trustworthy cooperator, especially in situations of tension or conflict. As Stephen Carter points out, ‘[s]haking hands traditionally signaled a lack of aggression. The open palm holds no weapon, and, while locked with someone else’s, cannot draw one’.^127^ Within the context of international diplomacy, disruptions to this practice can have significant implications. Carter continues,

Consider the iconic 1993 photograph of Yasser Arafat and Yitzhak Rabin shaking hands at the White House to symbolize their agreement to the Camp David Accords. Around the world, the image was cited as evidence that the violent standoff in the Middle East would finally change. The handshake mattered precisely because it was so hard to believe it had happened. Socially distance the two leaders and the photograph becomes incomprehensible, signaling nothing in particular.^128^

Due to public health considerations, politicians and diplomats have put the handshake on hold, as they navigate new ways to signal trust and cooperation with actions such as a friendly elbow bump.^129^ However, as Carter points out, ‘[b]umping fists or elbows cannot carry the same signal’.^130^ It is difficult to predict whether we will gradually witness a new diplomatic etiquette emerge in the international sphere, where gestures such as elbow bumps become normalized, or whether, as Carter seems to suggest, there will be ‘a bifurcated future…in which traditional manners continue fading from popular use but survive in such specialized arenas as business and international relations’.^131^

Second, social distancing is forcing diplomatic relationships to move online. As stated by Maricela Muñoz, Minister Counsellor at the Permanent Mission of Costa Rica to the United Nations in Geneva,

diplomats are facing multiple challenges, which encompass a broad range of elements, including access to adequate technology, the re-shaping of communication protocols, and the need to continue their work in virtual negotiation settings. The impossibility to meet face to face, given the confinement, social-distancing, and other sanitary measures, has demanded an overnight transformation of the diplomatic business as we know it.^132^

Shortened meetings and time zone differences are posing special challenges to diplomatic relationships. These include, for example, the difficulty of including technical experts and representatives from civil society, as well as a growing need for diplomats to ‘think on their feet’. Furthermore, it has often proved difficult to include delegates from developing countries due to technological and staffing issues. Some proposed solutions include a greater focus on permanent delegations at multilateral hubs like Geneva, New York, and Vienna; improving access to technological platforms and working procedures; and expanding diplomats’ skill sets, including their ability to interact with others in virtual environments.^133^

## Cover for Impolite Behaviour

The final problem we address in this chapter concerns the (mis)use of COVID-19 in order to provide an excuse for impolite behaviour. Even outside times of crisis, people often try to justify their (or others’) impolite behaviour by deflecting responsibility to their personality,^134^ communication style,^135^ or ‘personal frustrations and system failures’.^136^ Like overt breaches of politeness norms, these more covert forms of impoliteness can also create tensions and undermine social cooperation, for example in the workplace, where impoliteness can result in very real financial effects.^137^ Within the context of COVID-19, this problem has been exacerbated by the disruption of (and uncertainty surrounding) politeness norms that we have highlighted throughout this chapter. This has often contributed to facilitating disinhibitions, therefore reducing barriers to what might have previously been considered impolite behaviours.

### Personality and Impoliteness During COVID-19

Personality type can play a significant role in the way people experience the pandemic. Extroverts have had an especially tough time in social isolation under stricter lockdown orders.^138^ For example, one woman who self-identifies as an extrovert described her experience in this way:

That’s what the doctor told me: ‘go out once a week…go out somewhere and even have friends over but stay on the opposite side of the yard’ or things like that for my mental health because I am a people person. And it’s really getting me down. You know, it’s very difficult [to be an extrovert during the pandemic].^139^

Conversely, COVID-19 has been described as ‘an introverts dream’ in that it can provide introverts with an excuse for avoiding social situations and for ‘flaking’ on other people, which is admittedly an instance of rude behaviour.^140^ Even before the pandemic, appealing to one’s introverted personality in order to justify rude behaviour was not unusual. In a 2016 article in *The New York Times*, for example, writer and editor KJ Dell’Antonia admitted: ‘I’ve started to develop [a suspicion] that my introversion is an excuse for something else. I’m shy, yes. But am I also rude? In a contest between my manners and my preferences, am I allowing my preferences to win?’.^141^ While this rhetorical question suggests that introversion can indeed be used by some as an excuse for impolite or overtly rude behaviour, we should also consider other factors involved. First, some people genuinely have little control over their anti-social tendencies, and expectations around social behaviour can be anxiety-inducing. But, more importantly, the impoliteness for which introverts are often criticized may actually have positive implications. More specifically, one of the reasons why there is often a tension between an introverted personality and compliance with norms of politeness is the fact that those norms, and social institutions more generally, are grounded in (and tend to reward) extroverted behaviour.^142^ In other words, the problem might be structural, not (only) agential: if the rules of the game are set up in line with extroverts’ interests and preferences, then it will be inevitably more difficult for introverts to comply with them and be polite. Furthermore, this structural bias can have significant negative repercussions for introverts’ lives and opportunities. For example, research findings have shown that introverts generally earn less than extroverts^143^ and that they tend to be neglected or stigmatized in educational contexts.^144^

By bringing the tension between introversion and established politeness norms to the forefront of public debate, COVID-19 may actually provide an opportunity for critical reflection on existing social structures. This might promote a better understanding of the relationship between mental health and social institutions; create greater awareness of the distinct challenges that introverts and extroverts are experiencing during the pandemic^145^; and, more generally, encourage societies to embrace a more diverse approach to politeness. The latter point is especially important. In the same way multiculturalism theorists have demanded fairer terms of integration for minorities,^146^ scholars of civility (and the public more generally) may need to question the adoption of uniform norms of civility as politeness that disregard the many ways in which individuals and groups differ in terms of identity, personality, and social roles. This kind of critical judgment and reflection, as we already know, is itself central to what being polite means. Relatedly, and in line with the idea of ‘incivility as dissent’,^147^ some impolite behaviour during COVID-19 can be perceived as a challenge to unjust or unfair norms of politeness around which social structures and institutions have been shaped.

### Public Officials and Covert Impoliteness During COVID-19

In addition to ordinary citizens, the uncertainty concerning norms of politeness during COVID-19 has also been exploited by political figures as a cover for impolite behaviour. Here, though, it is important to distinguish between mere impoliteness and the kind of moral incivility that we will examine in the next chapter. The latter includes such instances of incivility as racist and sexist speech, which constitute a denial of their targets’ free and equal civic status. Conversely, impoliteness only involves an infringement on politeness norms, which are not tied to citizens’ free and equal status per se.

Returning again to the issue of wearing a mask, Donald Trump used impolite remarks more than once against the Democratic Party presidential candidate Joe Biden for wearing a mask in public. For example, in September 2020 he mocked Biden for letting his mask hang off his ear while delivering speeches, saying: ‘[d]id you ever see a man who likes a mask as much as him?…He has it hanging down. Because it gives him a feeling of security. If I were a psychiatrist, right, you know I’d say: “This guy’s got some big issues. Hanging down. Hanging down”’.^148^ On another occasion, he ridiculed Biden by asking: ‘What the hell did he spend all that money on the plastic surgery if he’s going to cover it up [with a mask]?’.^149^ One might argue that even in the absence of COVID-19 Trump would have probably found other ways to mock his rival. Yet, COVID-19 provided him and others with further opportunities for mocking, which in this example are closely related to the politicization of mask wearing in the US.

Here, we should take care to clarify the context of Trump’s remarks. Despite his initial scepticism regarding the importance of wearing a mask at the onset of the pandemic, Trump gradually accepted the overwhelming evidence provided by the scientific community regarding the importance of masks for containing the virus.^150^ However, Trump also stressed that in his view the appropriateness of mask wearing depends on context. For example, during a visit to Walter Reed Army Medical Center (WRAMC) in July 2020, he declared: ‘I think when you’re in a hospital, especially in that particular setting, where you’re talking to a lot of soldiers and people that, in some cases, just got off the operating tables, I think it’s a great thing to wear a mask…I’ve never been against masks, but I do believe they have a time and a place’. On another occasion, he said: ‘I don’t know, somehow, sitting in the Oval Office behind that beautiful Resolute Desk — the great Resolute Desk — I think wearing a face mask as I greet presidents, prime ministers, dictators, kings, queens, I don’t know. Somehow, I don’t see it for myself’.^151^

These statements are particularly interesting as they reveal two distinct aspects of Trump’s approach to mask wearing. One is the appropriateness of masks from a public health perspective: Trump agrees that people should generally wear them in some crowded places, especially hospitals. The other concerns norms of politeness and decorum: according to Trump, there are places and situations, such as official meetings with other heads of state, when wearing a mask is inappropriate, i.e. does not conform with etiquette and protocol. In such cases, Trump seems to argue, it is impolite to wear masks, even though this would be the appropriate thing to do from a public health perspective. On another occasion, Trump seemed to conflate the two dimensions, when he said the following about Biden: ‘Joe Biden can wear a mask, but he was standing outside with his wife, perfect conditions, perfect weather… And so I thought it was very unusual that he had one on. But I thought that was fine. I wasn’t criticizing at all. Why would I ever do a thing like that?’.^152^ Here Trump seems to hint at the inappropriateness of wearing a mask from both a politeness and a public health perspective—this was *both* a low-risk public health situation *and* one in which decorum and etiquette would have required Biden not to wear a mask. The main problem is that, due to the ambiguity of statements like these, some might understand Trump as implying that it is *always* safe not to wear a mask in non-hospital contexts. As a participant in one of Trump’s political rallies stated:

I don’t believe in the mask. That’s all…I don't care. I just don’t believe in it. Because I don’t understand what good it’s really going to do, unless you’re in a high-risk area like a nursing home or a hospital. I think the left is playing the mask thing to the hilt all the way to the election. In my opinion, they’re trying to steal the election.^153^

The foregoing analysis illustrates the uncertainty and disagreement regarding norms of politeness surrounding mask wearing during COVID-19. On the one hand, there are norms of politeness and etiquette that tell us we should be wearing a mask in public. These norms, like many general etiquette norms (e.g. sneezing into a tissue or your elbow, using a napkin rather than licking your fingers, flushing the toilet after using it) are grounded in public health evidence, e.g. preventing the spreading of germs and viruses.^154^ On the other hand, there are norms of politeness and etiquette (e.g. concerning diplomacy and formal political occasions) that are unrelated to public health but which, according to Trump, should also be taken into account when deciding whether to wear a mask or not. Trump exploited this context of uncertainty and disagreement, to which he himself had contributed, in order to cover for his impolite behaviour towards Biden and other mask-wearing US citizens.

The Australian politician Pauline Hanson provides another example. As a member of the Australian Senate, and leader of the right-wing populist One Nation party, Hanson has often been a vocal critic of immigration and multiculturalism in Australia. It could be argued that COVID-19 has provided Hanson with cover and opportunity to speak in negative terms about populations she often disparages in a way that is perhaps more widely accepted given the circumstances. For example, when in early July 2020 a sudden and strict stay-at-home order was imposed on 3,000 tenants in nine housing commission towers in Melbourne in order to contain a COVID-19 outbreak, Hanson referred to the tenants as ‘[t]hese people who cannot speak English, don’t know what the hell to do and they are actually then, spreading it to other people’.^155^ Setting aside the allegations of racism that followed Hanson’s statement,^156^ we suggest that COVID-19 provided cover for the impolite tone and delivery of her statements.

A final example concerns public officials, more specifically police officers in Australia. Speaking in early September 2020 about planned anti-lockdown protests by some Melbourne residents, Victoria Police’s Assistant Commissioner Luke Cornelius referred to them in the following way: ‘[t]he tin-foil hat-wearing brigade are alive and well out there in our community…I mean it’s just crazy. It’s bat s*** crazy nonsense’.^157^ We contend that this is impolite language that a high-ranking police officer would not use in ordinary circumstances, when it would be perceived as inappropriate by the vast majority of the public. However, the disruption of politeness norms resulting from COVID-19 has rendered this kind of language more acceptable. But unlike the previous examples concerning Trump and Hanson, we believe that Cornelius’s language can be defended. Its purpose was not to mock or ridicule certain individuals or categories of citizens per se (something which, especially in the Hanson example, closely resembles hate speech, an aspect of moral incivility to which we will return in the next chapter) but rather to disparage their decision to mobilize against public health efforts. In this sense, the use of impolite language by public figures to counter self-interested behaviour and promote public-minded goals could be considered a ‘top-down’ counterpart to the aforementioned ‘incivility as dissent’ paradigm. In the same way in which citizens can use incivility as impoliteness to signal and contest unjust laws and institutions from the bottom-up, public officials can use impolite messages from the top-down in order to foster just policy goals and norms and to hinder unjust ones, as long as these messages target certain people because of their actions rather than because of who they are (e.g. their ethnicity, religion, nationality, etc.).

### Name-Calling and Insults in International Politics During COVID-19

A final example of the use of COVID-19 to cover for impolite behaviour concerns name-calling and insults in international politics. Consider again Trump’s and other politicians’ decision to refer to COVID-19 as the ‘Wuhan virus’ or the ‘Chinese virus’. In addition to undermining international cooperation, as we saw in the previous section, the use of this kind of terminology highlights Trump’s use of COVID-19 as a cover for impolite language that breaches international norms of etiquette and protocol. Such norms certainly admonish name-calling and insults. Given the ongoing tensions between the US and China, one might argue that Trump used COVID-19 as a justification for using impolite language that he would have already preferred to use prior to the pandemic, in order to signal a stronger stance on China to his political base.

Here, however, we should be careful. Calling COVID-19 the ‘Wuhan virus’ or the ‘Chinese virus’ might be much more than impolite language. As many have pointed out, these statements may constitute instances of racist speech. Even though Trump stated that ‘[i]t’s not racist at all. No, not at all. It comes from China, that’s why. It comes from China. I want to be accurate’,^158^ various commentators have suggested that Trump’s statement may conceal a racist undertone. More specifically, according to some, the statement has contributed to an increase in anti-Chinese and anti-Asian racist incidents in the US, and can be traced back to a longstanding history of anti-Chinese sentiment in the US. For example, according to University of Minnesota professor Erika Lee,

[t]here is something very particular about the stereotype of China, Chinese people and Chinese faces as being unsanitary, teeming with millions of people living in crowded and dirty conditions, the weird habits that no civilized people would deign to follow, and Chinatowns as places of disease and contagion…Those stereotypes were used to justify quarantines and also immigration enforcement.^159^

If, as it seems plausible, these comments are correct, this example constitutes an instance of both impoliteness and moral incivility. Calling COVID-19 the ‘Wuhan virus’ and the ‘Chinese virus’ was both a breach of norms of international etiquette and protocol and an instance of anti-Chinese racism. The latter is not simply impolite. As we pointed out in Chapter 10.1007/978-981-33-6706-7_2, and will discuss more extensively in the next chapter, racist speech is an instance of moral incivility, which involves failing to treat others as free and equal. And, in relation to the problem examined in this section, it can be argued that COVID-19 provided Trump and other politicians with an excuse and cover for both impoliteness and racism. The former may have been driven by the pre-existing political and economic tensions between the US and China; the latter by a more general anti-immigrant and anti-multiculturalism rhetoric.

## Conclusion

This chapter examined new problems that COVID-19 poses for the politeness dimension of civility. From its onset, the pandemic has disrupted many of the norms that regulate people’s polite interactions in their everyday lives, resulting in a series of unprecedented challenges. First, COVID-19 has called into question previous norms of politeness that were widely recognized, making it more difficult for people to know how to behave politely in different circumstances. Second, this uncertainty has often deprived people of unequivocal ready-made polite signals to communicate their respect and consideration to others; speech or behaviour that in the past may have been unambiguously perceived as a polite signal might now go awry during the pandemic. Third, when people no longer know for sure how to be polite, or their polite signals misfire, this can also pose an obstacle to cooperative social interactions. Finally, citizens and politicians might sometimes exploit the uncertainty surrounding norms of politeness to engage in what would normally be considered impolite behaviour.

Responding to these challenges is crucial if we want to safeguard politeness and its key contributions to social coexistence. Both governments and citizens, we have seen, can play important roles in this response. To start, policymakers can remedy the lack of clarity surrounding norms of politeness by disseminating information, including scientific findings, to improve polite behaviour. This might include new signs and tools aimed at fostering awareness of how to behave politely in everyday spaces, in ways that are consistent with public health goals. While many of these interventions must ultimately come from governments, businesses and citizens should also do their part and make a proactive effort to acquire (and help each other acquire) the knowledge necessary to be polite in this new social landscape. They might also need to rely on creativity to develop new politeness norms when required, as in the case of greetings. These combined efforts can also help reduce the chance of politeness signals going awry. Developing new guidelines for both offline and online polite interactions, for instance, can help ensure that those signals do not misfire. Finding alternatives to signalling tools that are no longer easily available can help as well, including greater and more creative use of verbal communication and body language to compensate for reduced face visibility and expressions resulting from mandatory mask wearing.

Furthermore, promoting politeness during the pandemic can also help to minimize the social tensions that can result from breaches of politeness norms. There is an important space for both governments and private actors (e.g. retail businesses, airlines, etc.) to use informal and formal tools to encourage polite behaviour and sanction noncompliance. Within the international realm, adherence to norms of decorum and etiquette (sometimes adjusted to take into account public health goals) should be accompanied by the use of institutions to mitigate the challenges to cooperation posed by impolite behaviour. Finally, the pandemic might also help governments and citizens to develop a more critical and inclusive approach to politeness. They might even go so far as to recognize that impoliteness too can sometimes be employed by policymakers to promote public-minded policy goals, as when public officials rudely chastise those who refuse to comply with mask-wearing requirements.

### Notes

1. Katy Steinmetz, ‘How Do You Tell Others to Observe Social Distancing Rules?’, *Time*, 13 April 2020. https://time.com/5819816/coronavirus-social-distancing/.
2. Matteo Bonotti et al., ‘COVID-19 in Everyday Spaces: Social and Political Considerations’, *ABC Religion & Ethics*, 17 June 2020. https://www.abc.net.au/religion/covid19-in-everyday-spaces-and-social-division/12365294.
3. Gregory Rodgers, ‘Mosque Etiquette for Southeast Asia Visitors’, *TripSavvy*, 26 June 2019. https://www.tripsavvy.com/mosque-etiquette-for-visitors-1629901.
4. Gregory Rodgers, ‘Buddhist Temple Etiquette for Tourists: Dos and Don’ts’, *TripSavvy*, 15 September 2020. https://www.tripsavvy.com/visiting-buddhist-temples-dos-and-donts-1629907.
5. Anonymous, ‘Mass Etiquette’, *Parramatta Latin Mass Chaplaincy*, 2020. https://fssp-parra.org/mass-etiquette/.
6. Christina Ausley, ‘Seattle Farmers Markets to Reopen This Weekend with New “Market Manners”’, *KOMO*, 17 April 2020. https://komonews.com/news/local/seattle-farmers-markets-to-reopen-this-weekend-with-new-market-manners.
7. Anonymous, ‘Coronavirus Disease (COVID-19)’, *Government of Tasmania*, 8 April 2020. https://coronavirus.tas.gov.au/business-and-employees/covid-19-safe-workplaces-framework/guidance-for-retail-businesses.
8. Peta Fuller, ‘Will You Have to Queue for Easter Groceries? How New Supermarket Rules Will Work’, *ABC News*, 6 April 2020. https://www.abc.net.au/news/2020-04-06/coronavirus-supermarket-rules-coles-woolworths-aldi-add-queuing/12125656.
9. Anonymous, ‘Contact-Free Queue Management System for Social Distancing’, *Qminder*, 2020. https://www.qminder.com/social-distancing-queue-management/.
10. Alexis Dominguez, ‘Fight over Social Distancing at Colorado Springs Walmart Caught on Camera’, *KRDO*, 3 August 2020. https://krdo.com/news/2020/08/03/fight-over-social-distancing-at-colorado-springs-walmart-caught-on-camera/.
11. A US resident, video interview, 17 October 2020.
12. Kaitlyn Tiffany, ‘The Dos and Don’ts of “Social Distancing”’, *The Atlantic*, 12 March 2020. https://www.theatlantic.com/family/archive/2020/03/coronavirus-what-does-social-distancing-mean/607927/.
13. Gemima Cody, ‘Five Things All Good Diners Should Do When Restaurants Reopen’, *Good Food*, 30 May 2020. https://www.goodfood.com.au/eat-out/news/five-things-all-good-diners-should-do-when-restaurants-reopen-20200529-h1oe6d.
14. Micheline Maynard, ‘Dos and Don’ts for Restaurant Orders During The Coronavirus Crisis’, *Forbes*, 27 March 2020. https://www.forbes.com/sites/michelinemaynard/2020/03/27/dos-and-donts-for-restaurant-orders-during-the-coronavirus-crisis/#6b3eea7545f6.
15. Maynard, ‘Dos and Don’ts for Restaurant Orders During The Coronavirus Crisis.’.
16. Sam Wydymus, ‘In These Hard Times, Restaurant No Shows Aren’t Just Rude – They’re Fatal’, *The Guardian*, 18 July 2020. https://www.theguardian.com/commentisfree/2020/jul/18/restaurant-no-shows-politeness-uk-dining.
17. A Marketing and Functions Coordinator at a restaurant in Melbourne, interview questions via personal correspondence, 21 October 2020.
18. Lauren Sieben, ‘Tipping Etiquette in the Time of Coronavirus: How Much Is Enough?’, *SFGate*, 6 May 2020. https://www.sfgate.com/realestate/article/Tipping-Etiquette-in-the-Time-of-Coronavirus-How-15251571.php.
19. Rachel Hosie, ‘This Is How Much People in Different Countries Value Personal Space’, *The Independent*, 2 May 2020. https://www.independent.co.uk/life-style/personal-space-boundaries-different-countries-argentina-uk-romania-a7713051.html.
20. Emma Taggart, ‘Parisian Café Uses Giant Teddy Bears to Ensure Social Distancing’, *My Modern Met*, 29 June 2020. https://mymodernmet.com/paris-cafe-giant-teddy-bears-social-distancing/.
21. Monique Woo, ‘These Photos Provide a Glimpse into How Restaurants Are Reopening around the World’, *The Washington Post*, 13 June 2020. https://www.washingtonpost.com/travel/2020/06/13/these-photos-provide-glimpse-into-how-restaurants-are-reopening-around-world/.
22. Emma Taggart, ‘German Cafe Enforces Social Distancing Rules by Asking Customers to Wear Pool Noodle Hats’, *My Modern Met*, 18 May 2020. https://mymodernmet.com/german-cafe-pool-noodles/.
23. Natalia Liubchenkova, ‘In Pictures: Restaurants Find Creative Ways to Enforce Distancing’, *Euronews*, 20 July 2020. https://www.euronews.com/2020/07/20/in-pictures-restaurants-find-creative-ways-to-enforce-social-distancing.
24. Margaret Visser, *The Rituals of Dinner: The Origins, Evolution, Eccentricities, and Meaning of Table Manners* (Open Road Media, 2015).
25. Pip Sloan, ‘The Post-Lockdown Menu: Is This the End of Sharing Platters and Communal Seating?’, *The Telegraph*, 29 May 2020. https://www.telegraph.co.uk/food-and-drink/features/post-lockdown-menu-will-become-small-plates-sharing-platters/.
26. Li Lei and Zhang Hui, ‘COVID-19 Changes Chinese’ Hygiene, Dining Etiquette – Maybe Permanently’, *Global Times*, 20 May 2020. https://www.globaltimes.cn/content/1188896.shtml.
27. Kaitlin Woolley and Ayelet Fishbach, ‘Shared Plates, Shared Minds: Consuming from a Shared Plate Promotes Cooperation’, *Psychological Science*, Vol. 30, No. 4 (2019): 541–552. https://doi.org/10.1177/0956797619830633.
28. Alex-Louise Tessonneau, ‘Learning Respect in Guadeloupe: Greetings and Politeness Rituals’. In *Politeness and Face in Caribbean Creoles*, ed. Susanne Mühleisen and Bettina Migge (John Benjamins Publishing Company Amsterdam, 2005), 255–282.
29. Ashley Fetters, ‘When Keeping Your Distance Is the Best Way to Show You Care’, *The Atlantic*, 10 March 2020. https://www.theatlantic.com/family/archive/2020/03/how-coronavirus-caused-hug-and-handshake-hiatus/607762/.
30. Nick Duffy, ‘France Urges Kissing Ban to Stop the Spread of Coronavirus’, *Inews*, 29 February 2020. https://inews.co.uk/news/world/france-kissing-ban-coronavirus-covid-19-403456.
31. Mehmet Ozalp, ‘How Coronavirus Challenges Muslims’ Faith and Changes Their Lives’, *The Conversation*, 2 April 2020. https://theconversation.com/how-coronavirus-challenges-muslims-faith-and-changes-their-lives-133925.
32. A bank employee in Italy, interview questions via personal correspondence, 23 October 2020 (Translated from Italian into English by the authors).
33. Fetters, ‘When Keeping Your Distance Is the Best Way to Show You Care’.
34. Capricia Penavic Marshall, *Protocol: The Power of Diplomacy and How to Make It Work for You* (HarperCollins, 2020).
35. Anonymous, ‘“I Shook Hands with Everybody,” Says Boris Johnson Weeks before Coronavirus Diagnosis’, *The Guardian*, 27 March 2020. https://www.theguardian.com/world/video/2020/mar/27/i-shook-hands-with-everybody-says-boris-johnson-weeks-before-coronavirus-diagnosis-video.
36. Mark Sklansky, Nikhil Nadkarni, and Lynn Ramirez-Avila, ‘Banning the Handshake from the Health Care Setting’, *JAMA*, Vol. 311, No. 24 (2014): 2477–2478. https://doi.org/10.1001/jama.2014.4675.
37. Bjarke Oxlund, ‘An Anthropology of the Handshake’, *Anthropology Now*, Vol. 12, No. 1, published online on 25 June 2020: 39–44. https://doi.org/10.1080/19428200.2020.1761216.
38. David Beard, ‘Here Are Some Handshake Alternatives, as Suggested by Our Readers’, *History & Culture*, 17 March 2020. https://www.nationalgeographic.com/history/2020/03/handshake-alternatives-suggested-by-readers/.
39. Patrick Tardos, ‘How to Say Hello When We Can No Longer Shake Hands’, *The Daily Telegraph*, 18 May 2020. https://www.dailytelegraph.com.au/coronavirus/hibernation/how-to-say-hello-without-shaking-hands/news-story/f7eb723f4e60d7c82c6ed9348f7ede79; Caroline Davies, ‘Elbow-Bumps and Footshakes: The New Coronavirus Etiquette’, *The Guardian*, 3 March 2020. https://www.theguardian.com/world/2020/mar/03/elbow-bumps-and-footshakes-the-new-coronavirus-etiquette; Sophie Aubrey, ‘Hello “Foot-Shake”? The New Rules of Etiquette in a Time of Coronavirus’, *The Sydney Morning Herald*, 2 March 2020. https://www.smh.com.au/lifestyle/health-and-wellness/hello-foot-shake-the-new-rules-of-etiquette-in-a-time-of-coronavirus-20200302-p5462o.html.
40. Cheshire Calhoun, ‘The Virtue of Civility’, *Philosophy & Public Affairs*, Vol. 29, No. 3 (2000): 251–275. https://doi.org/10.1111/j.1088-4963.2000.00251.x.
41. Amy Irwin, ‘That’s Just Rude: Why Being Polite May Not Be a Universal Concept’, *The Conversation*, 4 April 2018. https://theconversation.com/thats-just-rude-why-being-polite-may-not-be-a-universal-concept-94187.
42. Justine Zhang et al., ‘Conversations Gone Awry: Detecting Early Signs of Conversational Failure’. In *Proceedings of the 56th Annual Meeting of the Association for Computational Linguistics*, Vol. 1 (2018): 1350–1361. https://doi.org/10.18653/v1/P18-1125.
43. Mark Andrew, ‘Japanese Commuters Are So Polite They Don’t Give Up Their Seats For The Elderly’, *Elite Readers*, 22 September 2015. https://www.elitereaders.com/japan-public-transportation-etiquette/; Anonymous, ‘Why Don’t the Japanese Give Priority to the Elderly on Public Transportation?’, *Japan Info*, 3 August 2020. https://jpninfo.com/21297.
44. Janice D. Yoder et al., ‘Exploring Moderators of Gender Differences: Contextual Differences in Door-Holding Behavior’, *Journal of Applied Social Psychology*, Vol. 32, No. 8 (2002): 1682–1686. https://doi.org/10.1111/j.1559-1816.2002.tb02769.x.
45. Of course, there is also the possibility that a man may hold the door for a woman *because* he expects her to perceive that act as sexist and he *wants* to be sexist.
46. Larry David, ‘Type + Distance = No Door Hold’, *Curb Your Enthusiasm*, Season 9, Episode 1 (2017). https://www.youtube.com/watch?v=CyFSAzwLtPk&ab_channel=JohnnyWestside.
47. Michelle Cleary and Adele Freeman, ‘Email Etiquette: Guidelines for Mental Health Nurses’, *International Journal of Mental Health Nursing*, Vol. 14, No. 1 (2005): 62–65. https://doi.org/10.1111/j.1440-0979.2005.00356.x.
48. Anna K. Turnage, ‘Email Flaming Behaviors and Organizational Conflict’, *Journal of Computer-Mediated Communication*, Vol. 13, No. 1 (2007): 43–59. https://doi.org/10.1111/j.1083-6101.2007.00385.x.
49. Jim Herrera, ‘I Hope This Email Finds You Well’, *The New York Times*, 19 August 2020. https://www.nytimes.com/2020/08/19/smarter-living/coronavirus-how-to-write-better-emails.html.
50. Some of these recommendations also apply, of course, to email communication with family members and friends.
51. Clarissa Sebag-Montefiore, ‘How to Write Emails in a Pandemic’, *BBC*, 8 June 2020. https://www.bbc.com/worklife/article/20200604-whats-the-right-way-to-sign-off-emails-during-coronavirus.
52. John Montogery, ‘Video Meeting Etiquette: 7 Tips to Ensure a Great Attendee Experience’, *Zoom Blog*, 27 November 2020. https://blog.zoom.us/video-meeting-etiquette-tips/.
53. Bryan Lufkin, ‘The Zoom Social Etiquette Guide’, *BBC*, 29 April 2020. https://www.bbc.com/worklife/article/20200428-the-zoom-social-etiquette-guide.
54. Anonymous, ‘Zoom Meetings: Etiquette and Best Practices’, *University of Pittsburgh*, 26 March 2020. https://www.technology.pitt.edu/blog/zoom-tips.
55. Erin Wen Ai Chew (Founder and National Convener for the Asian Australian Alliance), video interview, 21 October 2020.
56. Simon Kolstoe, ‘Coronavirus: Wearing a Cloth Face Mask Is Less about Science and More about Solidarity’, *The Conversation*, 20 May 2020. https://theconversation.com/coronavirus-wearing-a-cloth-face-mask-is-less-about-science-and-more-about-solidarity-138461.
57. Megan Garber, ‘Refusing to Wear a Mask Is an Empty Act of Defiance’, *The Atlantic*, 27 May 2020. https://www.theatlantic.com/culture/archive/2020/05/face-mask-videos-culture-wars-trump-logic/612139/.
58. Ryan Lizza and Daniel Lippman, ‘Wearing a Mask Is for Smug Liberals. Refusing to Is for Reckless Republicans.’, *Politico*, 1 May 2020. https://www.politico.com/news/2020/05/01/masks-politics-coronavirus-227765.
59. Alejandra Borunda, ‘Americans Are Wearing Masks Now, and Their Meaning Is Changing’, *National Geographic*, 27 April 2020. https://www.nationalgeographic.com/science/2020/04/coronavirus-america-face-mask-culture-changing-meaning-changes-too/.
60. Bianca Nobilo, ‘Coronavirus Has Stolen Our Most Meaningful Ways to Connect’, *CNN*, 2020. https://www.cnn.com/interactive/2020/06/world/coronavirus-body-language-wellness.
61. Mark S. Nestor, Daniel Fischer, and David Arnold, ‘“Masking” Our Emotions: Botulinum Toxin, Facial Expression, and Well-Being in the Age of COVID-19′, *Journal of Cosmetic Dermatology*, Vol. 19, No. 9 (2020): 19. https://doi.org/10.1111/jocd.13569.
62. A woman in Italy, interview questions via personal correspondence, 22 October 2020 (Translated from Italian into English by the authors).
63. Medgadget editors, ‘CIVILITY Mask Lets People See Each Others’ Faces During Pandemic’, *Medgadget*, 22 June 2020. https://www.medgadget.com/2020/06/civility-mask-lets-people-see-each-others-faces-during-pandemic.html.
64. Peggy Drexler, ‘How to Read Body Language in the Era of Face Masks’, *British Vogue*, 6 September 2020. https://www.vogue.co.uk/beauty/article/body-language-face-masks.
65. Thomas Shambler, ‘People Are Turning to Unusual and Inventive Masks to Beat COVID-19”, *Esquire Middle East*, 24 March 2020. https://www.esquireme.com/content/44720-people-are-turning-to-unusual-and-inventive-masks-to-beat-covid-19.
66. Anonymous, ‘Masks, Identity and Bias’, *Anti-Defamation League*, 2020. https://www.adl.org/media/14554/download.s.
67. Adam Bloodworth, ‘How Face Masks Became a Powerful Symbol of Expression in Dark Times’, *HuffPost Australia Life*, 26 August 2020. https://www.huffingtonpost.com.au/entry/coronavirus-face-masks-powerful-symbol_au_5f2cdf8ec5b6e96a22b050b3.
68. Rachel Tashjian, ‘Your Mask Is Now Your Political Identity’, *GQ*, 4 May 2020. https://www.gq.com/story/mask-coronavirus-politics.
69. Carolyn Meynes, ‘Coronavirus Etiquette: Face Masks, Working from Home and More’, *The Active Times*, 18 May 2020. https://www.theactivetimes.com/home/coronavirus-etiquette-face-mask-work-from-home/slide-20.
70. Felix Bathon, ‘Holding Doors for Others — A History of the Emergence of a Polite Behavior’, *InterDisciplines. Journal of History and Sociology*, Vol. 9, No. 2 (2018). https://doi.org/10.4119/indi-1074.
71. Zizi Papacharissi, ‘Democracy Online: Civility, Politeness, and the Democratic Potential of Online Political Discussion Groups’, *New Media & Society*, Vol. 6, No. 2 (2004): 259–283. https://doi.org/10.1177/1461444804041444.
72. Marina Terkourafi, ‘Conventionalization: A New Agenda for Im/Politeness Research’, *Journal of Pragmatics*, Vol. 86 (2015): 11–18. https://doi.org/10.1016/j.pragma.2015.06.004.
73. Bonotti et al., ‘COVID-19 in Everyday Spaces: Social and Political Considerations’.
74. An academic from Israel, personal correspondence, 11 March 2020.
75. Sofia Quaglia, ‘Exercise in the Time of Coronavirus: How to Work out Safely in a Pandemic’, *Inverse*, 13 March 2020. https://www.inverse.com/mind-body/exercise-coronavirus-how-to-workout-safely-in-a-pandemic.
76. Example Derek Edyvane, ‘Incivility as Dissent’, *Political Studies*, Vol. 68, No. 1 (2019): 93–109. https://doi.org/10.1177/0032321719831983.
77. Richard Boyd, ‘“The Value of Civility?”’, *Urban Studies*, Vol. 43, No. 5–6 (2006): 871. https://doi.org/10.1080/00420980600676105.
78. Sune Laegaard, ‘Religious Neutrality, Toleration and Recognition in Moderate Secular States: The Case of Denmark’, *The Ethics Forum*, Vol. 6, No. 2 (2011): 86. https://doi.org/10.7202/1008033ar.
79. Chris Cillizza, ‘The Merkel-Trump Handshake Heard’round the World’, *CNN*, 6 July 2020. https://www.cnn.com/2017/07/06/politics/merkel-trump-handshake/index.html.
80. Louis Nelson, ‘Trump Struggles with Group Handshake at ASEAN Meeting’, *Politico*, 13 November 2017. https://www.politico.com/story/2017/11/13/trump-handshake-asean-asia-meeting-244835.
81. Brenna Williams, ‘Donald Trump Shook the Japanese Prime Minister’s Hand for 19 s’, *CNN Politics*, 13 February 2017. https://edition.cnn.com/2017/02/10/politics/trump-abe-awkward-diplomacy/index.html.
82. Simon Osborne, ‘Donald Trump: The REAL Reason I Didn’t Shake Hands with Angela Merkel’, *Express*, 14 November 2017. https://www.express.co.uk/news/world/879411/Donald-Trump-Angela-Merkel-handshake.
83. Cillizza, ‘The Merkel-Trump Handshake Heard’round the World’.
84. Trump and Merkel subsequently shook hands before a bilateral meeting preceding the G20 summit in Hamburg on 6 July 2017.
85. Emily Stewart, ‘Trump Walks in Front of the Queen, and Apparently That’s Not Allowed’, *Vox*, 14 July 2018. https://www.vox.com/policy-and-politics/2018/7/14/17571986/trump-walks-in-front-of-queen.
86. Meagan Fredette, ‘Trump Ends Tumultuous U.K. Visit With A Breach In Royal Protocol’, *Refinery29*, 15 July 2018. https://www.refinery29.com/en-us/2018/07/204376/trump-queen-england-royal-protocol-breach.
87. Anonymous, ‘Berlusconi Rebuke Makes Queen a YouTube Hit’, *Metro*, 3 April 2009. https://metro.co.uk/2009/04/03/berlusconi-rebuke-makes-queen-a-youtube-hit-602199/.
88. Arona Maskil, ‘Mistakes That Will Damage Your Business in the UAE’, *Globes*, 1 September 2020. https://en.globes.co.il/en/article-mistakes-that-will-damage-your-business-in-the-uae-1001341335.
89. U. S. Jolly, *Challenges for a Mega City: Delhi, a Planned City with Unplanned Growth* (Concept Publishing Company, 2010); Bill Luckin, ‘War on the Roads: Traffic Accidents and Social Tension in Britain, 1939–45’, *Accidents in History* (Brill Rodopi, 1997): 234–254.
90. Sandee LaMotte, ‘Road Rage Is on the Rise. Here’s How to Survive Dangerous Encounters.’, *CNN*, 10 September 2019. https://www.cnn.com/2019/09/10/health/road-rage-survival-tips-wellness/index.html.
91. Anonymous, ‘Good Driving Etiquette: Being Polite on the Road’, *Driver Knowledge Test Resources*, 9 December 2019. https://www.driverknowledgetests.com/resources/good-driving-etiquette-being-polite-on-the-road/.
92. Emanuela Ceva and Michele Bocchiola, ‘Theories of Whistleblowing’, *Philosophy Compass*, Vol. 15, No. 1 (2020). https://doi.org/10.1111/phc3.12642.
93. Bret D. Asbury, ‘Anti-Snitching Norms and Community Loyalty’, *Oregon Law Review*, Vol. 89, No. 4 (2009): 1257–1312. https://dx.doi.org/10.2139/ssrn.1491630; Malin Åkerström, ‘The Social Construction of Snitches’, *Deviant Behavior*, Vol. 9, No. 2 (1988): 155–167. https://doi.org/10.1080/01639625.1988.9967776.
94. Patrick Wood, ‘When Should You Call the Police about Alleged Coronavirus Restriction Breaches?’, *ABC News*, 18 July 2020. https://www.abc.net.au/news/2020-07-19/should-you-call-police-about-coronavirus-restriction-breaches/12460892.
95. Wood, ‘When Should You Call the Police about Alleged Coronavirus Restriction Breaches?’.
96. A Melbourne resident, interview questions via personal correspondence, 13 October 2020.
97. David Nitkin, ‘Becoming a Snitching Culture’, *EthicScan*, 7 May 2020. https://ethicscan.ca/blog/2020/05/07/becoming-a-snitching-culture/.
98. John MacFarlane, ‘Should You Snitch on Your Neighbours for Flouting Physical Distancing Rules? Here’s Some Advice’, *CBC News*, 28 March 2020. https://www.cbc.ca/news/canada/montreal/navigating-physical-distancing-rules-covid-19-etiquette-1.5513472.
99. MacFarlane, ‘Should You Snitch on Your Neighbours for Flouting Physical Distancing Rules? Here’s Some Advice’.
100. MacFarlane, ‘Should You Snitch on Your Neighbours for Flouting Physical Distancing Rules? Here’s Some Advice’.
101. Robin Young and Samantha Raphelson, ‘Miss Manners Updates Etiquette Guidelines For The COVID-19 Age’, *wbur*, 27 May 2020. https://www.wbur.org/hereandnow/2020/05/27/miss-manners-contagious-etiquette.
102. Anonymous, ‘Workplace Bullying on the Rise Due to Coronavirus Fears’, *DiversityQ*, 26 March 2020. https://diversityq.com/workplace-bullying-on-the-rise-due-to-coronavirus-fears-1509112/.
103. Anonymous, ‘Workplace Bullying on the Rise Due to Coronavirus Fears’.
104. A bank employee in Italy, interview questions via personal correspondence, 23 October 2020 (Translated from Italian into English by the authors).
105. Damon Cronshaw, ‘Supermarket Staff Copping Abuse and Aggression amid Coronavirus Tension’, *Newcastle Herald*, 17 March 2020. https://www.newcastleherald.com.au/story/6679938/supermarket-staff-copping-abuse-and-aggression-amid-coronavirus-tension/.
106. Cronshaw, ‘Supermarket Staff Copping Abuse and Aggression amid Coronavirus Tension’.
107. Amy Graff, ‘California Woman Allegedly Pees on Floor of Verizon Store after Being Asked to Wear a Mask’, *SFGate*, 21 July 2020. https://www.sfgate.com/bayarea/article/California-woman-pees-Verizon-store-no-mask-15423242.php.
108. Edyvane, ‘Incivility as Dissent’*.*
109. Francesca Street, ‘Is Covid-19 Making Airplane Passengers More Unruly?’, *CNN*, 13 August 2020. https://www.cnn.com/travel/article/unruly-airplane-passengers-covid-19/index.html.
110. Thomas Pallini, ‘See the Yellow Card Alaska Airlines Will Give to Passengers Who Refuse to Wear Masks on Its Flights’, *Business Insider*, 2 July 2020. https://www.businessinsider.com.au/alaska-giving-yellow-cards-to-flyers-who-dont-wear-masks-2020-7?r=US&IR=T.
111. Ben Doherty, ‘China and Australia: How a War of Words over Coronavirus Turned to Threats of a Trade War’, *The Guardian*, 3 May 2020. https://www.theguardian.com/australia-news/2020/may/03/china-and-australia-how-a-war-of-words-over-coronavirus-turned-to-threats-of-a-trade-war.
112. Doherty, ‘China and Australia: How a War of Words over Coronavirus Turned to Threats of a Trade War’.
113. Nathan Vanderklippe, ‘With Angry Words around the World, China Uses COVID-19 Pandemic to Take on Superpower Mantle’, *The Globe and Mail*, 30 April 2020. https://www.theglobeandmail.com/world/article-with-angry-words-around-the-world-china-uses-covid-19-pandemic-to/.
114. Holger Limberg, ‘Impoliteness and Threat Responses’, *Journal of Pragmatics*, Vol. 41, No. 7 (2009): 1376–1394. https://doi.org/10.1016/j.pragma.2009.02.003; Holger Limberg, ‘Threats in Conflict Talk: Impoliteness and Manipulation’, *Impoliteness in Language: Studies on Its Interplay with Power in Theory and Practice* (2008): 155–180. https://doi.org/10.1515/9783110208344.3.155.
115. Doherty, ‘China and Australia: How a War of Words over Coronavirus Turned to Threats of a Trade War’.
116. Anonymous, ‘COVID-19: Canada Immigration FAQs and News’, *Canadavisa*, 21 October 2020. https://www.canadavisa.com/coronavirus-covid-19-impact-canada-immigration-visa-border-latest-news.html.
117. Gavin Moodie, ‘Coronavirus: How Likely Are International University Students to Choose Australia over the UK, US and Canada?’, *The Conversation*, 22 July 2020. https://theconversation.com/coronavirus-how-likely-are-international-university-students-to-choose-australia-over-the-uk-us-and-canada-142715.
118. John Daley and Will Mackey, ‘Australian Unis May Need to Cut Staff and Research If Government Extends Coronavirus Travel Ban’, *The Conversation*, 20 February 2020. https://theconversation.com/australian-unis-may-need-to-cut-staff-and-research-if-government-extends-coronavirus-travel-ban-132175.
119. Zhaoyin Feng, ‘Being a Chinese Student in the US: “Neither the US nor China Wants Us”’, *BBC News*, 3 August 2020. https://www.bbc.com/news/world-us-canada-53573289.
120. John Ross, ‘China Warns Australia of Student Boycott’, *Times Higher Education*, 28 April 2020. https://www.timeshighereducation.com/news/china-warns-australia-student-boycott.
121. Lorraine Brown, ‘International Education: A Force for Peace and Cross‐cultural Understanding?’, *Journal of Peace Education*, Vol. 6, No. 2 (2009): 209–224. https://doi.org/10.1080/17400200903086672.
122. David Choi, ‘G7 Countries Fail to Deliver a Joint Statement Because US Insists on Saying “Wuhan Virus” for the Coronavirus’, *Business Insider Australia*, 26 March 2020. https://www.businessinsider.com.au/g7-failed-to-reach-agreement-because-of-wuhan-virus-2020-3.
123. Luke Moffett, ‘Why Calls for Reparations from China for Coronavirus Are an Unfeasible Distraction’, *The Conversation*, 9 June 2020. https://theconversation.com/why-calls-for-reparations-from-china-for-coronavirus-are-an-unfeasible-distraction-139684; Grady McGregor, ‘Trump’s Suggestion That China Pay Coronavirus Reparations Evokes an Ugly History’, *Fortune*, 8 March 2020. https://fortune.com/2020/05/07/trump-china-pay-coronavirus-reparations/.
124. Reinhard Wolf, ‘Respect and Disrespect in International Politics: The Significance of Status Recognition’, *International Theory*, Vol. 3, No. 1 (2011): 105–142. https://doi.org/10.1017/S1752971910000308.
125. Arthur A. Stein, ‘Neoliberal Institutionalism’. In *The Oxford Handbook of International Relations*, ed. Christian Reus-Smit and Duncan Snidal (New York: Oxford University Press, 2008), 201–221.
126. Rachel Gray, ‘The Way Forward for Australia-China Trade Relations’, *UNSW Sydney Newsroom*, 3 July 2020. https://newsroom.unsw.edu.au/news/general/way-forward-australia-china-trade-relations.
127. Stephen L. Carter, ‘How COVID-19 Is Killing Good Manners’, *The Japan Times*, 26 September 2020. https://www.japantimes.co.jp/opinion/2020/09/26/commentary/world-commentary/covid-19-killing-good-manners/.
128. Carter, ‘How COVID-19 Is Killing Good Manners’.
129. Caroline Davies, ‘Elbow-bumps and Footshakes: The New Coronavirus Etiquette’, *The Guardian*, 3 March 2020. https://www.theguardian.com/world/2020/mar/03/elbow-bumps-and-footshakes-the-new-coronavirus-etiquette.
130. Carter, ‘How COVID-19 Is Killing Good Manners’.
131. Carter, ‘How COVID-19 Is Killing Good Manners’.
132. Maricela Muñoz, ‘Diplomacy in Times of COVID-19’, *DiPLO*, 16 July 2020. https://www.diplomacy.edu/blog/diplomacy-times-covid-19.
133. Muñoz, ‘Diplomacy in Times of COVID-19’.
134. Arpan Bhattacharya, ‘Is Introversion an Excuse for Rudeness? Here’s How to Self-Correct and Flourish’, *Big Think*, 27 September 2016. https://bigthink.com/arpan-bhattacharyya/introversion-vs-rudeness; Randy Conley, ‘Your Personality Is Not An Excuse For Bad Behavior’, *Leading with Trust*, 20 May 2012. https://leadingwithtrust.com/2012/05/20/your-personality-is-not-an-excuse-for-bad-behavior/.
135. Amy Morin, ‘Why You Can’t Afford To Ignore Rude Behavior - Workplace Incivility Costs You More Than You Think’, *Forbes*, 12 August 2016. https://www.forbes.com/sites/amymorin/2016/08/12/why-you-cant-afford-to-ignore-rude-behavior-workplace-incivility-costs-you-more-than-you-think/.
136. Anonymous, ‘Disrespectful Behaviors: Their Impact, Why They Arise and Persist, and How to Address Them (Part II)’, *Institute For Safe Medication Practices*, 24 April 2014. https://www.ismp.org/resources/disrespectful-behaviors-their-impact-why-they-arise-and-persist-and-how-address-them-part.
137. Morin, ‘Why You Can’t Afford To Ignore Rude Behavior - Workplace Incivility Costs You More Than You Think’.
138. Brittany Wong, ‘How Introverts And Extroverts Handle The COVID-19 Pandemic Differently’, *Huffington Post*, 10 September 2020. https://www.huffingtonpost.com.au/entry/introverts-extroverts-covid-pandemic_l_5f57bbbdc5b646e336620ef3.
139. A US resident, personal video interview, 17 October 2020.
140. Pallavi Pundir, ‘As an Introvert, Coronavirus Is a Perfect Excuse for My Social Distancing’, *Vice*, 14 March 2020. https://www.vice.com/en/article/z3b85x/as-an-introvert-coronavirus-is-a-perfect-excuse-for-my-social-distancing.
141. K.J. Dell’Antonia, ‘Am I Introverted, or Just Rude?’, *The New York Times*, 24 September 2016. https://www.nytimes.com/2016/09/25/opinion/sunday/am-i-introverted-or-just-rude.html.
142. Bhattacharya, ‘Is Introversion an Excuse for Rudeness?’.
143. Molly Owens, ‘Personality Type & Career Achievement: Does Your Type Predict How Far You’ll Climb?’, *Truity Psychometrics LLC*, February 2015. https://www.truity.com/sites/default/files/PersonalityType-CareerAchievementStudy.pdf.
144. Susan Cain, ‘How Do Teachers Feel about Their Quiet Students?’, *Quiet Revolution*, 23 October 2015. https://www.quietrev.com/how-do-teachers-feel-about-their-quiet-students/.
145. Mark Travers, ‘Are Extroverts Suffering More From The Quarantine? Not So Fast, Says New Research’, *Forbes*, 30 April 2020. https://www.forbes.com/sites/traversmark/2020/04/30/are-extroverts-suffering-more-from-the-quarantine-not-so-fast-says-new-research/#249d432851a0; Brittany Wong, ‘How Introverts And Extroverts Handle The COVID-19 Pandemic Differently’, *Huffington Post*, 9 September 2020. https://www.huffpost.com/entry/introverts-extroverts-covid-pandemic_l_5f57bbbdc5b646e336620ef3.
146. Will Kymlicka, *Multicultural Citizenship: A Liberal Theory of Minority Rights* (Clarendon Press, 1995).
147. Edyvane, ‘Incivility as Dissent’.
148. Anonymous, ‘The Latest: Trump Mocks the Way Biden Wears His Mask’, *AP NewsS*, 4 September 2020. https://apnews.com/article/7d093d8fefee24b97f449f83d405c9dd.
149. John T. Bennett, ‘Trump Mocks Biden for Wearing a Face Mask: “What Was the Plastic Surgery For?”’, *The Independent*, 22 September 2020. https://www.independent.co.uk/news/world/americas/us-election/url-trump-biden-covid-masks-plastic-surgery-b540606.html.
150. Anonymous, ‘CDC Calls on Americans to Wear Masks to Prevent COVID-19 Spread’, *Centers for Disease Control and Prevention*, 14 July 2020. https://www.cdc.gov/media/releases/2020/p0714-americans-to-wear-masks.html.
151. Melanie Mason, ‘The Presidential Cover-up, or How Trump Went from Shunning to Wearing a Mask’, *Los Angeles Times*, 14 July 2020. https://www.latimes.com/politics/story/2020-07-14/trump-coronavirus-mask-evolution.
152. Chris Cillizza, ‘Analysis: Donald Trump’s Latest Attack on Mask-Wearing May Be His Worst Yet’, *CNN*, 4 September 2020. https://www.cnn.com/2020/09/04/politics/donald-trump-joe-biden-masks/index.html.
153. Kadia Goba and Matt Berman, ‘Trump Made Fun of Politicians Wearing Masks During a Crowded Rally Where Many Didn’t Wear Masks’, *BuzzFeed News*, 3 September 2020. https://www.buzzfeednews.com/article/kadiagoba/trump-coronavirus-masks-campaign-rally.
154. Anonymous, ‘How Rude’, *The Emily Post Institute*, n.d. https://emilypost.com/advice/how-rude/; Hannah Smothers, ‘Wearing a Mask Is Simply Good Manners’, *Vice*, 9 July 2020. https://www.vice.com/en/article/5dzyed/wearing-a-mask-is-simply-good-manners.
155. Anonymous, ‘One Nation’s Pauline Hanson Launches Attack on Melbourne Housing Commission Tenants Locked Down after COVID Outbreak’, *PerthNow*, 6 July 2020. https://www.perthnow.com.au/news/coronavirus/one-nations-pauline-hanson-launches-attack-on-melbourne-housing-commission-tenants-locked-down-after-covid-outbreak-ng-b881599640z.
156. Naaman Zhou and Margaret Simons, ‘Today Show Dumps Pauline Hanson for “divisive” Remarks about Melbourne Public Housing Residents’, *The Guardian*, 6 July 2020. https://www.theguardian.com/australia-news/2020/jul/06/today-show-dumps-pauline-hanson-for-divisive-remarks-about-melbourne-public-housing-residents.
157. Lucy Mae Beers, ‘Top Victoria Police Officer Slams Melbourne Anti-Lockdown Stage 4 Protesters’, *7NEWS*, 28 August 2020. https://7news.com.au/news/victoria-police/top-victoria-police-officer-slams-melbourne-anti-lockdown-stage-4-protesters--c-1274007.
158. Quint Forgey, ‘Trump on “Chinese Virus” Label: “It’s Not Racist at All”’, *Politico*, 18 March 2020. https://www.politico.com/news/2020/03/18/trump-pandemic-drumbeat-coronavirus-135392.
159. Caitlin Yoshiko Kandil, ‘Asian Americans Report over 650 Racist Acts over Last Week, New Data Says’, *NBC News*, 27 March 2020. https://www.nbcnews.com/news/asian-america/asian-americans-report-nearly-500-racist-acts-over-last-week-n1169821.

